# Cleaner production technologies for the amelioration of soil health, biomass and secondary metabolites in *Ocimum basilicum* L. under Indian Western Himalaya

**DOI:** 10.3389/fpls.2022.976295

**Published:** 2022-11-09

**Authors:** Yog Raj, Nilofer Ali, Aparna Maitra Pati, Rakesh Kumar

**Affiliations:** ^1^ Agrotechnology Division, CSIR-Institute of Himalayan Bioresource Technology, Palampur, India; ^2^ Biotechnology Division, CSIR-Institute of Himalayan Bioresource Technology, Palampur, India; ^3^ Academy of Scientific and Innovative Research (AcSIR), Ghaziabad, India

**Keywords:** biostimulants, essential oil, leaf gaseous exchange, methyl chavicol, plant growth promoting rhizobacteria, soil health, sustainable production

## Abstract

*Ocimum basilicum* L. and its derived products are primarily consumed by humans; hence, agrochemical use seems inappropriate for its cultivation. However, farmers are accustomed to using rampant inorganic fertilizers to augment crop productivity, which has unintendedly engendered severe environmental perturbations. Concomitantly, farmers will soon have to confront the challenges of growing crops under suboptimal conditions driven by global climate change. Consequently, to develop a clean, sustainable, and resilient production technology, field experiments spanning over two years (2020 and 2021) were conducted, comprising three biostimulants, *viz*., vermicompost (0, 4, and 8 Mg ha^−1^), biofertilizer (uninoculated and inoculated), and liquid seaweed extract (without and at 7 ml L^−1^) in the Indian western Himalaya for the first time. Soil health indicators, leaf photosynthetic pigments, gaseous exchange, mineral contents, essential oil (EO) quantity, and composition were evaluated. Soil microbial respiration (SMR), microbial biomass carbon (MBC), organic carbon (OC), dehydrogenase (DHA), alkaline phosphatase (ALP), and β-glucosidase activities were increased by 36.23, 83.98, 30.61, 42.69, 34.00, and 40.57%, respectively, when compared with the initial soil status. The net photosynthetic rate (Pn) was significantly increased with the highest (8 Mg ha^−1^) and moderate (4 Mg ha^−1^) vermicompost dosages by 13.96% and 4.56%, respectively, as compared with the unfertilized control (0 Mg ha^−1^). Likewise, the biofertilizer and seaweed extract also enhanced Pn by 15.09% and 10.09%, respectively. The crop’s key EO constituents, *viz*., methyl chavicol and linalool, were significantly improved with the highest and moderate vermicompost rates of 2.71, 9.85%, and 1.18, 5.03%, respectively. Similarly, biofertilization and seaweed application also boosted methyl chavicol and linalool by 3.29, 8.67%, and 1.93, 3.66%, respectively. In both years, significantly higher herbage (8.86 and 11.25 Mg ha^−1^) and EO yield (113.78 and 154.87 kg ha^−1^) were recorded with a congregate treatment of the highest vermicompost dose, biofertilizer, and liquid seaweed extract. In conclusion, the integrated use of biostimulants having complementary properties can sustainably maximize the quantity and quality of *O. basilicum* and concomitantly ameliorate soil health. This study can inspire scientific communities and industries to develop second-generation biostimulant products, delivering better sustainability and resilience for a renaissance in agriculture.

## Introduction

Global consumption of primary nutrient fertilizers, *viz*., nitrogen (N), phosphate (P_2_O_5_), and potash (K_2_O) applied in agriculture is expected to increase annually by ~1.5, 2.2, and 2.4%, respectively, and eventually reach up to ~202 million Mg by the end of 2020 (FAO, 2017). India, being the largest consumer of chemical fertilizers, consumes ~16% N, ~19% P_2_O_5_, and ~15% K_2_O nutrients every year compared to the total world’s consumption (Parween et al., 2017). Although chemical fertilizers augment crop productivity, their extensive and imprudent use has unfortunately prompted soil health degradation (Banerjee et al., 2019), eutrophication, climate change (Frank et al., 2019), and consequently declined agricultural productivity (Adegbeye et al., 2019). In addition, shortages of synthetic chemical fertilizers like urea, ammonium nitrate, calcium ammonium nitrate, mono, di-ammonium phosphates, and single, triple-superphosphates are also expected in the near future, as they are all made from non-renewable resources (Blouin et al., 2019). Concomitantly, their production process also emits harmful greenhouse gases (GHG_S_) like methane (CH_4_), oxides of carbon (CO and CO_2_), nitrogen (NO_x_), and sulfur (SO_2_), even a poisonous volatile hydrogen fluoride gas (HF) (Hao et al., 2020). Meanwhile, ~80% and 25%–75% of N and P_2_O_5_ fertilizers get lost in the environment, and this loss is encouraged by excessive precipitation/rainfed agroecosystems (Choudhary and Kumar, 2019). Likewise, ~1% of chemical pesticides reach their target sites, and the remaining amount resides in the environment. These worries about using synthetic inorganic agrochemicals compelled scientific communities to develop alternative, environment-friendly, and safer methods to improve crop growth and agricultural yields in a sustainable manner. In this context, organic farming, which defies and abates agrochemical use, has sparked exorbitant consumer attention and scientific interest. Nonetheless, the main hitch in organic farming is its lower and erratic yield compared to conventional farming. The judicious application of biostimulants, which are compatible with organic farming, can act as a panacea, truncating this apparent yield gap between organic and conventional agriculture systems. Moreover, global climate change has also aggravated irregularities in crop yields. This could be reconciled with the use of biostimulants, as they can regulate and alter the plant’s physiological processes to confront or alleviate stressors and consequently assure yield stability.

Sweet basil (*Ocimum basilicum* L.) is an annual, essential oil (EO) producing medicinal aromatic plant (MAP), predominantly cultivated for direct human consumption and chemical use is inappropriate for its production (Maham et al., 2018). The crop ranks second after spearmint; methyl chavicol and linalool are the key constituents attributed to the crop’s characteristic aroma (Tahami et al., 2017). It is cultivated during the rainy season in tropical areas throughout the world. India has occupied ~60% (3,000 ha) of the world’s area (5,000 ha), generating ~70% of EO (350 Mg) annually, contrary to the total world’s production (~500 Mg) which is still short for its growing demand (Absar et al., 2016). Furthermore, globally, ~3,000 forms of plant EO are traded (~40,000–60,000 Mg annum^−1^) with an estimated commercial market value of ~US $700 million which is also forecasted to expand in the coming years (Chouhan et al., 2019). The basil EO market is also estimated to increase with a 6.2% cumulative average growth rate (CAGR), thereby reaching a value of US$ 285.69 million in 2027. Sweet basil EO is widely used either directly as methyl chavicol or after its conversion into anethole in the food and flavoring industries for the preparation of baked goods, condiments, vinegars, oils, cheese, jams, teas, ice creams, sausages, meat products, salad dressings, liqueurs, and beverages. Some pharmaceutical industries also use basil EO for making gripe water. Furthermore, for millennia, people have used basil leaves and their EO to treat several common ailments like headaches, colds, digestive problems, and bloating. Also, in the conventional medicine system, basil is prescribed as a stimulant, fever reducer, antimalarial, and anti-flatulent (Tavallali et al., 2018). Meanwhile, the worldwide basil leaf market is also predicted to rise with a 1.3% CAGR, reaching up to 62 million by 2026 (Sipos et al., 2021), thus indicating its significant consumption.

Considering the perils of using inorganic fertilizers and the worth of MAPs, the use of organic biostimulants, *viz*., compost, biofertilizers, and seaweed extracts can be recommended as an alternative solution for their sustainable production (Bhattacharyya et al., 2021; Gupta et al., 2021a; Huang et al., 2021; Kulkarni et al., 2021). These can provide almost all essential nutrients for crop growth and also provide micronutrients, *viz*., copper (Cu), iron (Fe), zinc (Zn), and manganese (Mn) at optimal levels with better acquisition. Additionally, these can supply organic carbon (OC), which improves soil edaphic conditions and ultimately increases crop yield. Among composts, vermicompost is considered the best fertilizer, having higher humic substances (HSs), being homogenous, microbiologically active, and less phytotoxic (Soobhany, 2019). Moreover, vermicomposting is a greener and cleaner process that involves the bio-oxidation and stabilization of organic waste due to the interactions between earthworms and microorganisms. Likewise, nowadays propensity of using biofertilizers is prevailing as a suitable alternative to counteract the adverse environmental impacts exerted by synthetic agrochemicals (Gupta et al., 2021b). They are composed of microbial living cells which can augment plant growth by making some of the vital nutrients available from their unavailable forms through regulation of biogeochemical cycles (C, N, P, K, S), production of plant growth regulators (PGRs) like indole-3-acetic acid (IAA), gibberellic acid (GA), and cytokinin (CK), and discharge of biological materials such as vitamin B, nicotinic acid, pantothenic acid, and biotin into the soil (Mącik et al., 2020). Additionally, the seaweed extracts possess macro and micronutrients including some PGRs such as IAA, CK, abscisic acid, amino acids, and vitamins which can improve plant growth, soil microecology, and fertilizer use efficiency indicating it as a potential fertilizer/biostimulant (Jung and Kim, 2020). Besides these, a recent study claimed that the presence of some secondary metabolites in seaweed extracts like sulfabenzamide, 1-phosphatidyl-1D-myoinositol, and dodecanamide has proven bioactivities related to plant growth promotion and disease resilience (Vaghela et al., 2022). Also, the presence of quaternary ammonium compounds like glycine, betaine, and choline chloride in seaweed extracts may impart resilience to crops against various stressors, especially under rainfed conditions because of their osmolytic properties (Singh et al., 2016b). Furthermore, the production process of seaweed extract is eco-friendlier and does not entail any harmful field emissions (Singh et al., 2018; Sudhakar et al., 2019).

Owing to the plant growth promoting (PGP) characteristics and biostimulatory response of vermicompost, biofertilizer, and seaweed extracts (Wozniak et al., 2020), we hypothesized that their congregate use could remarkably augment sweet basil productivity compared to sole application and also increase EO synthesis holistically by altering the physiological and biochemical processes of the plant. As plant growth-promoting rhizobacteria (PGPR) present in biofertilizers are heterotrophic in nature and continuously require a source of carbon and energy, adding vermicompost can decisively augment PGPR growth and metabolic activities by providing readily available nutrients, eventually leading to enhanced crop productivity. Concomitantly, the seaweed extract can promote the growth of PGPR and also establish unison with their host plant by inducing a mimicking effect. Furthermore, the current prevailing voyage of investigating the complementary properties of applying combinations of different biostimulant categories has demonstrated their additive/synergistic effect on improving crop productivity (Gupta et al., 2021a; Sani and Yong, 2021). While, some combination treatments like PGPR (*Pseudomonas fluorescens* and *Bacillus licheniformis*) and smoke-derived compound (karrikinolide) showed antagonistic effects also (Papenfus et al., 2015; Salomon et al., 2017). Nevertheless, these investigations were predominantly performed on non-MAP crops like onion, groundnut, grapevine, lettuce, okra, etc., under controlled/semi-controlled conditions; albeit, their evaluations under open field conditions are scanty and remain a discerning task (Rouphael and Colla, 2018; Rouphael and Colla, 2020). As crops confront combined multifaceted stressors in open fields, which are difficult to mimic in a controlled environment; also, the biostimulants screened in controlled environments do not always enact as expected under field conditions (Rouphael et al., 2018). Moreover, field-scale evaluation of biostimulants on growth, productivity, secondary metabolite accumulation, and composition, especially in MAPs, is still a major backdrop.

Based on the available literature, the synergistic and additive properties between biostimulant categories are fascinating and indicative of their intricate biostimulatory mechanisms in determining plant growth, performance, and resilience. Consequently, it is indispensable to decipher the sole and combined effects of biostimulants to invent the next generation of biostimulant products having harmonious properties for improved crop growth, yield, quality, and resilience. Additionally, with improved mechanistic clarity, it will be possible to design deliberate combinations of non-microbial and microbial biostimulants that would interact synergistically to provide the required results in terms of acceptable yield and quality products in a sustainable manner. Therefore, accentuating the inextricable and serious collateral problems of using chemical fertilizers, coupled with their forthcoming shortages, and the context of sustainable production of MAPs, the current study was designed to evaluate the synergistic/additive effect of diverse biostimulants on growth, productivity, secondary metabolite accumulation, and composition in sweet basil. The salient objectives of the study were: (i) to analyze the effect of biostimulants on the physiological performance of sweet basil under field conditions; (ii) to elicit the amount and key constituents of sweet basil’s EO and elucidate its plausible mechanism; and (iii) to evaluate the effect of organic biostimulant amendments on soil physical, chemical, and biological properties.

## Materials and methods

### Experimental details, cultivation, and treatment procedures

A field study was conducted for two consecutive cropping years (2020 and 2021) at the experimental farm of CSIR-Institute of Himalayan Bioresource Technology, Palampur, Himachal Pradesh, India (elevation: 1,328 m above mean sea level; latitude: 32°11′39′′ N; longitude: 76°56′51′′ E). The first factor was composed of three levels of vermicompost (0, 4, and 8 Mg ha^−1^); the second factor was comprised of two levels of biofertilizer (uninoculated, or inoculated), and the third factor consisted of two levels of seaweed extract (control, and foliar application at 7 ml L^−1^) (**Table 1**). The experimental plot consisted of three blocks, each 25 m long and contained 12 plots of 3.6 m × 3.0 m. In each plot, 48 plants were grown within 45 cm × 50 cm spacing, eight rows of 6 plants plot^−1^. The composition and attributes of biostimulants used in the current study are provided in **Table 2**.

**Table 1:** Abbreviation and treatment details.

Sr. No.	Treatment code	First factor (organic manure)	Second factor (biofertilizer)	Third factor (sea weed extract)	Abbreviation
1	T1	No added manure	−	−	V−
2	T2	No added manure	−	+	S+
3	T3	No added manure	+	−	B+
4	T4	No added manure	+	+	B+S+
5	T5	Vermicompost at 4 Mg ha**^−^** ^1^	−	−	V4
6	T6	Vermicompost at 4 Mg ha**^−^** ^1^	−	+	V4S+
7	T7	Vermicompost at 4 Mg ha**^−^** ^1^	+	−	V4B+
8	T8	Vermicompost at 4 Mg ha**^−^** ^1^	+	+	V4B+S+
9	T9	Vermicompost at 8 Mg ha**^−^** ^1^	−	−	V8
10	T10	Vermicompost at 8 Mg ha**^−^** ^1^	−	+	V8S+
11	T11	Vermicompost at 8 Mg ha**^−^** ^1^	+	−	V8B+
12	T12	Vermicompost at 8 Mg ha**^−^** ^1^	+	+	V8B+S+

V, vermicompost; V−, unfertilized control; V4, vermicompost at 4 Mg ha**^−^** ^1^; V8, vermicompost at 8 Mg ha**^−^** ^1^; B, biofertilizer; S, seaweed extract; (−), without factor; (+), with factor.

**Table 2:** Physicochemical attributes and composition of organic manure (vermicompost), carrier material (biochar), biofertilizer, and liquid seaweed extract (Sagarika, IFFCO, India) used during the experiment.

^#^Organic manure (Vermicompost)													
Attributes		pH	EC (m mhos cm^−1^)	OC (%)	N (%)			P (%)	K (%)				
Value		6.9 ± 0.10	7.1 ± 0.20	27.53 ± 0.16	1.49 ± 1.21			0.41 ± 0.33	2.30 ± 0.60				
^#^Carrier material (biochar)													
Attributes		pH	EC (m mhos cm^−1^)	OC (%)	Bulk density (Mg cm^−3^)				WHC (%)				
Value		6.1 ± 0.12	3.6 ± 0.13	5.30 ± 0.23	0.71 ± 0.41				8.9 ± 0.19				
^#^Biofertilizer													
*Arthrobacter psychrochitiniphilus* IHB B 13602 (KU160185)						*Bacillus altitudinis* IHBT 705 (CP074101)							
PGP attributes	PS (µg ml^−1^)		IAA (µg ml^−1^)	SP (mm)	ACCD activity		PS (µg ml^−1^)			IAA (µg ml^−1^)	SP (mm)		ACCD activity
Value	62.82 ± 1.03		27.30 ± 1.21	20 ± 1.32	+		66.25 ± 1.01			59.91 ± 1.17	15 ± 0.92		+
^*^Seaweed extract (Sagarika, IFFCO, India; 28% w/w *Kappaphycus alvarezii* and *Sargassum swartzii*)													
Attributes	N (%)		P (%)	K (%)	S (%)			Auxin (ppm)	Cytokinin (ppm)			Gibberellin (ppm)	
Value	0.12−0.30		0.05−0.19	14.00−18.00	1.25−2.25			400−600	200−400			500−800	

^#^Results of composition of organic manure (vermicompost), and carrier material (biochar) and biofertilizer attributes are presented as means of three replicates (n = 3) ± standard deviation.

^*^Whereas the composition of seaweed extract was provided by the manufacturer (IFFCO, India) on the label of the product.

pH, potential of hydrogen; EC, electrical conductivity; OC, organic carbon; N, available nitrogen; P, available phosphorous; K, available potassium; S, available sulfur; WHC, water holding capacity; PGP attributes, plant growth promoting attributes; PS, inorganic phosphate solubilization; IAA, indole-3-acetic acid production; SP, siderophore production (qualitative); ACCD, 1-aminocyclopropane-1-carboxylate deaminase activity.

The Indian sweet basil seeds of accession IHBT/OC-1, having 98% vigor, were manually planted in pots comprising sand, soil, and farmyard manure in a 1:1:1 ratio within a depth of 1–2 cm during the first week of June in both years. Seeds were germinated 7–14 days after sowing, and thirty-day-old seedlings (30 DOS) having an average root length of 11.93 cm and a shoot length of 15.76 cm (**Supplementary Figure S1A**) were used for transplantation. The corresponding dose of vermicompost was added by manually spreading it on the topsoil surface of the corresponding plots 30 days before transplanting. The biofertilizer was prepared with two PGPRs strains, i) *Arthrobacter psychrochitiniphilus* IHB B 13602 (KU160185) isolated from the rhizosphere of agricultural land situated at the cold desert region of the Lahaul valley and ii) *B. altitudinis* IHBT 705 (CP074101) harnessed from the native agricultural field of Palampur, Himachal Pradesh, India. These PGPR strains were selected based on their multiple PGP traits like IAA synthesis (27.30 ± 1.21, 59.91 ± 1.17 µg ml^−1^), siderophore production (20.00 ± 1.32, 15.00 ± 0.92 mm), phosphate solubilization (62.82 ± 1.03, 66.25 ± 1.01 µg ml^−1^), and ACCD (1-aminocyclopropane-1-carboxylate deaminase) activity (**Table 2**). The preparation of biofertilizer was carried out by growing them in sterile tryptone soya broth (TSB) at 28°C (180 rpm; 24 h). Afterward, the bacterial cells were pelleted down by centrifugation at 6,000 rpm by using an Eppendorf 5804R benchtop refrigerated centrifuge for 10 min, followed by dissolution into sterile deionized water and mixing with a sterile carrier material (biochar). Thereafter, to make a consortium, both were mixed at a 1:1 ratio; the biofertilizer thus formed has 1.8 × 10^8^ CFU ml^−1^ bacterial cells. Biofertilizer was applied at two stages: i) at the seedling transplanting stage (30 DOS; 6–8 leaves stage); ii) at the first weeding event; 20 days after transplanting (DAT) after the establishment of plants in the main field.

**Figure 1:**
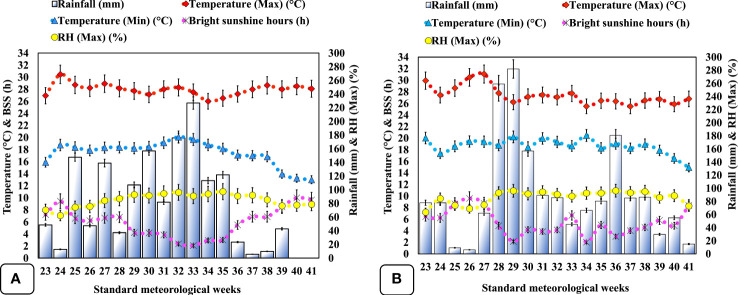
Climatogram representing weekly mean meteorological data during the period of the experiment (2020–2021) at Palampur, Himachal Pradesh, India. **(A)** weather data of the first cropping year (June–November, 2020); **(B)** weather data of the second cropping year (June-November, 2021). BSS, bright sunshine hours; RH (Max), maximum relative humidity.

The first inoculation of biofertilizer was achieved through root dipping (Verma et al., 2016) of disinfected seedlings (**Supplementary Figure S1B**) for 15 min; sterile jaggery was used as a sticking agent, whilst booster inoculation was facilitated through a hoeing procedure in well-established plants by exposing the rhizospheric region of each plant and stuffing it with ~5 g biofertilizer, followed by covering it with soil to increase the bacterial rate and to ensure the infection of the new roots (Anli et al., 2020). Foliar application of liquid seaweed extract (28% w/w; Sagarika, IFFCO, India) derived from *Kappaphycus alvarezii* (red algae) and *Sargassum swartzii* (brown algae) at 7 ml L^−1^ was done using a Knapsack sprayer with a constant flow until complete canopy runoff during a cloudless sunny day (Elansary et al., 2016) at vegetative (15 DAT) and flower initiation stage (40 DAT). The crop was completely grown under rainfed conditions, with only two intermittent hand-weeding events at 20 and 35 DAT, respectively. The soil of the experimental area was characterized as silty clay with low OC (0.49 ± 0.11%), acidic pH (5.3 ± 0.02), deficient in P_2_O_5_ (7.60 ± 0.87 kg ha^−1^), moderate in available N (139.10 ± 2.39 kg ha^−1^), and rich in available K_2_O (318.00 ± 3.19 kg ha^−1^) (**Supplementary Table S1**). The climate of the region was subtropical. Weekly mean weather conditions like rainfall, temperature (maximum and minimum), relative humidity, and bright sunshine hours for the entire experimental duration were retrieved from the crop weather outlook agro-meteorological advisory (Anonymous, 2022) and have been represented in **Figure 1**. All considered climatic conditions varied during both experimental years, and notably, total rainfall was ~16.83% higher in the second year (**Figure 1B**) as compared with the first cropping season (**Figure 1A**).

### Assessment of soil health

Soil samples before and after the experiment were collected from each plot with five core points (0–30 cm depth) and the rhizosphere of five plants from each plot, respectively. At each sampling event, 10–20 g of soil was collected, and the specimens were mixed to get a composite sample depicting the whole field. Soil biological, physicochemical, and enzymatic properties were analyzed. To assess the biological features such as soil microbial respiration (SMR), microbial biomass carbon (MBC), bacterial population count (BPC), and soil enzymatic activities some soil subsamples were put in zip-lock plastic bags and immediately kept inside a cold room (8°C), rest samples were shade dried, sieved with a 2 mm mesh and kept at room temperature until analysis. The OC was evaluated by adding potassium dichromate solution (K_2_Cr_2_O_7_; 10 ml, 0.5 M), and deionized water (150 ml) to 0.5 g of homogenized soil. Diphenylamine was added followed by titration using 1 N ammonium iron (III) sulfate hexahydrate and expressed in percentage (OC%). The soil texture was determined by the hydrometer method while, pH was detected with soil and water (1:2) suspension by a glass electrode pH meter. A macro Kjeldahl method was adopted for the determination of available N by digesting 0.5 g of soil in a Kjeldahl digestion tube with concentrated sulfuric acid (10 ml), and the N concentration was determined by an N analyzer (Kelplus classic DX VA, Pelican Instruments Pvt. Ltd., India). Available phosphorous (P) was determined with a soil solution extracted with 0.5 M sodium bicarbonate (NaHCO_3_) at 470 nm using a spectrophotometer (T90+ UV/vis, PG Instruments Limited, UK). At the same time, the amount of exchangeable K was quantified with 1 N extractable ammonium acetate (NH_4_OAc) by flame atomic emission spectrometry (Raj et al., 2021). Soil micronutrients *viz*., Mn, Cu, Zn, and Fe were quantified using an atomic absorption spectrophotometer as per the standard protocols (Jones, 1998), while the BPC was performed by serial dilution up to 10 folds by taking 1 g of soil into a sterile test tube containing 9 ml of sterile 0.9% normal saline and expressed as CFU g^−1^ soil (Raj et al., 2019).

The MBC and SMR were quantified using a modified fumigated and alkali absorption extraction techniques, respectively (Dehsheikh et al., 2020). Uniformly, soil (5 g) was fumigated with chloroform at 27°C for 24 h, followed by the addition of potassium sulfate (0.5 M; 25 ml) to make a soil suspension. The suspension was agitated for 30 min, and then filtered, followed by pH calibration (6.5–8.5). Afterwards, 10 ml of potassium dichromate (0.05 M) and 200 ml of deionized water were added, followed by titration with ferric ammonium sulfate hexahydrate (0.5 N) and diphenylamine. Simultaneously, the same procedure was repeated in non-fumigated soils as well. In addition, the soil dry weight was determined by placing 5 g of soil inside a hot air oven at 105°C. The MBC was estimated as per the standard equation and expressed as mg C 100 g^−1^ dry soil. To determine SMR, 10 g of homogenized soil was dispersed in a glass beaker having a lid, and the tube containing 5 ml of NaOH (0.25 M) solution was placed inside each flask to capture the CO_2_, followed by incubation at 27°C for 48 h. The remaining NaOH solution was titrated with HCl (0.25 M), BaCl_2_ (0.5 M), and phenolphthalein. Simultaneously, 10 g of soil was dried at 105°C, and SMR was calculated using a standard equation and expressed as mg CO_2_ g^−1^ dry soil day^−1^.

Soil enzymatic activities like dehydrogenase (DHA), urease, alkaline phosphatase (ALP), and β-glucosidase were accessed as per the standard procedures (Mukherjee et al., 2021) with appropriate modifications. DHA was estimated by taking uniformly soil (5 g) in a 50 ml flask, followed by the addition of calcium carbonate (0.1 g), 3% triphenyl tetrazolium chloride (TTC), and 4 ml of deionized water. The suspension was mixed gently by tapping and allowed to incubate at 37°C for 24 h. Then, for extraction, 40 ml of acetone was added. This was followed by the mixing of the content and then filtering. Furthermore, additional acetone was added and the volume was raised to 50 ml. Optical density (OD) was taken at 485 nm using a spectrophotometer for triphenyl formazan (TPF) in samples using a standard calibration curve and DHA was expressed as μg TPF g^−1^ dry soil h^−1^. For determination of urease activity, soil (5 g) was taken in a flask (100 ml) and 2.5 ml of urea solution (0.08 M) was poured over. Then, the flask was stoppered and allowed to incubate at 37°C for 2 h, followed by the addition of 50 ml of potassium chloride solution (1 N KCl in 0.01 N HCl) and vigorously shaken for 30 min. Then the solution was filtered, and the clean filtrate (1 ml) was pipetted into another flask (50 ml), followed by the addition of deionized water (9 ml), sodium salicylate/sodium hydroxide solution (5 ml), and 2 ml of sodium dichloro-isocyanurate solution. The contents of flasks were incubated at room temperature for 30 min, followed by quantification of ammonium (NH_4_⁺) using a spectrophotometer at 690 nm, and urease activity was expressed as μg N-NH_4_^+^ g^−1^ dry soil h^−1^.

For determination of ALP uniformly, 1 g of soil was taken in a flask (50 ml), followed by the addition of 0.25 ml toluene, 4 ml modified universal buffer (MUB) (pH 11), and 1 ml p-nitrophenyl phosphate solution (PNP). The flask was stoppered, followed by the mixing of all contents, and allowed to incubate at 37°C for 1 h. Afterward, 0.5 M calcium chloride (1 ml) and 0.5 M sodium hydroxide (4 ml) were added and again mixed to form a suspension. The suspension was then filtered, the filtrate’s OD was measured at 400 nm, and the amount of soil ALP activity was expressed as μg PNP g^−1^ dry soil h^−1^. The β-glucosidase activity was assessed by taking uniformly soil (1 g) in a test tube followed by the addition of toluene (0.25 ml). Thereafter, 4 ml of MUB (pH 6) and 1 ml of p-nitrophenyl-β-D-glucoside (25 mM PNG) were added, followed by proper mixing. The suspension was incubated at 37°C for 1 h, followed by the addition of 1 ml of calcium chloride (0.5 M) and tris solution (4 ml) followed by filtration, and OD was taken at 400 nm. A calibration curve of standard p-nitrophenyl-β-D-glucoside (PNG) was prepared and the activity was expressed as μg PNG g^−1^ dry soil h^−1^.

### Plant sampling

Non-peripheral net plants (22) were considered and selected for various observations in all treatments to avoid the border effect. Five plants from each plot were tagged for observation of growth attributes like plant height, plant spread, and primary branches at 50 and 100 DAT. Leaf, stem, and inflorescence weight, number of inflorescences, average length of inflorescence, herbage yield, EO content, and yield were recorded at harvest. In addition, a few healthy leaves were also taken at 50 and 100 DAT to determine the photosynthetic pigments and leaf mineral concentration, respectively.

### Estimation of photosynthetic pigments, mineral nutrients, and gaseous exchange

Uniformly, 1 g of fresh leaf sample was washed with deionized water. It was followed by homogenization with 80% acetone and centrifugation at 6,000×*g* for 5 min to collect the supernatant. The OD of the collected supernatant was measured at 663, 646, and 470 nm for Chl_a_, Chl_b_, and carotenoids, respectively, using a spectrophotometer. Acetone (80%) was used as a blank for the procedure, and concentrations of Chl_a_, Chl_b_, and carotenoids (mg g^−1^) were calculated according to formulas previously described by Rathore and Kumar (2021). Additionally, dried pulverized leaves (0.5 g) were digested with concentrated H_2_SO_4_ and a mixture of concentrated H_2_SO_4_:HClO_4_ (5:1) for estimation of N and P, respectively. The contents of N were estimated with an N analyzer while P was quantified by a spectrophotometer.

Three individual plants were selected from each plot for the measurement of gas exchange-associated characteristics like net photosynthetic rate (P_n_), stomatal conductance (G_s_), transpiration rate (T_r_), intracellular CO_2_ (C_i_), and leaf vapor pressure deficit (VPD_leaf_) using a portable leaf chamber of 2 cm × 3 cm (6 cm^2^) having a red‐blue LED light source and an infrared gas analyzer (LICOR-6400 XT^®^, LI‐COR Biosciences, Lincoln, NE, USA). Before all measurements, the instrument was warmed up for ~30 min and an auto program was run to determine the light dependence of СО_2_ gaseous exchange in leaves with ten log points, and a light intensity curve was established by giving PAR ranging from 0 to 2,000 μmol photons m^−2^ s^−1^ (**Supplementary Figure S2**). During both years, physiological parameters were measured during the flower initiation stage every day from 9:00 am to 11:00 am when the sky was clear. The third fully expanded leaf was placed inside the chamber by ensuring it was touched with a thermocouple from the underside. Chamber conditions were set as CO_2_ at 400 mmol mol^−1^ provided by a 12 g CO_2_ cartridge (LI-COR Bioscience, Lincoln, NE, U.S.A.) using a CO_2_ mixer with a flow rate of 500 μmol s^−1^, relative humidity of 50%–60%, and PAR was set at 1,300 mmol m^−2^ s^−1^ as determined by light calibration curve (**Supplementary Figure S2**). The block temperature and average leaf area were set at 25°C and 2.5 cm^2^, respectively. Data were considered and logged when flow rate, CO_2_, and H_2_O were stable to quantify the leaf gaseous exchange parameters. Additionally, intrinsic water use efficiency (WUE_int_) and the ratio of intracellular CO_2_ to ambient CO_2_ were calculated as P_n_/T_r_ and C_i_/C_a_, respectively.

### Essential oil extraction and yield determination

A uniform weight (1,000 g) of 24 h shade-dried aboveground parts (leaf and inflorescence) excluding stems from every plot was hydro-distilled for 3 h in the Clevenger apparatus. The extracted EO was treated with anhydrous sodium sulfate (Na_2_SO_4_) and kept in a sealed amber glass vial at 4°C for further analysis. The EO weight (g 1,000^−1^g) of aboveground dried parts was used to compute the EO content (% w/w). The EO yield was estimated using the following formula and expressed in kg ha^−1^.


EO yield=herbage yield×EO content×specific gravity (0.9)×10 (conversion factor).

### Quantification of essential oil constituents

Quantitative evaluation of EO compounds was done by taking three biological replicates with three technical repeats on a single quadrupole gas chromatograph-mass spectrometer (GC-MS) through a flame ionization detector (FID) (GC QP2010 SE, Shimadzu Corp., Tokyo, Japan) fitted with an AOC 5000 Plus auto-injector comprised of Zebron ZB-5 MS capillary column (length: 30 m; internal diameter: 0.25 mm; thickness: 0.25 μm). The extracted EO (10 μl) was dissolved in dichloromethane (2 ml), followed by injection in split mode (2 μl each). Nitrogen gas was used as a carrier at a 1.5 ml min^−1^ flow rate. The temperature was set to 70°C for 3 min, then increased at a rate of 4°C min^−1^ for 5 min, with injector and detector temperatures of 280°C and 300°C, respectively. The mass spectrometer was operated at 70 eV ionization energy and the reading speed (50–500 m/z) was set at 1 scan^−1^. The GC peak was used to compute the number of volatile constituents and their amounts, which were then sorted by the order of GC elution. The retention index (RI) was calculated without correction factors using a series of hydrocarbons. The constituents of EO were recognized by a comparison of the experimental RI_s_ with RI_s_ described in the literature (Adams, 2017). Additionally, the constituents were also recognized by comparing the components’ lowest mass spectral fragmentation pattern with the NIST library (Stein, 2005).

### Statistical analysis

Datasets of two years were pooled and used to evaluate the effects of the cropping year and used biostimulants on growth, yield, EO amount and composition, leaf nutrient uptake, and gaseous exchange using a four-factor analysis of variance (ANOVA). The results were represented as the mean of three replications (*n = 3*) ± SE. Data were analyzed by comparing means using Fisher’s LSD (least significant difference) at a *P = 0.05* confidence level (SYSTAT Software Inc., Chicago, Illinois, USA). A co-relationship between crop traits was also determined by using Pearson’s coefficient (PAST-4).

## Results

### Soil health condition

**Figure 2:**
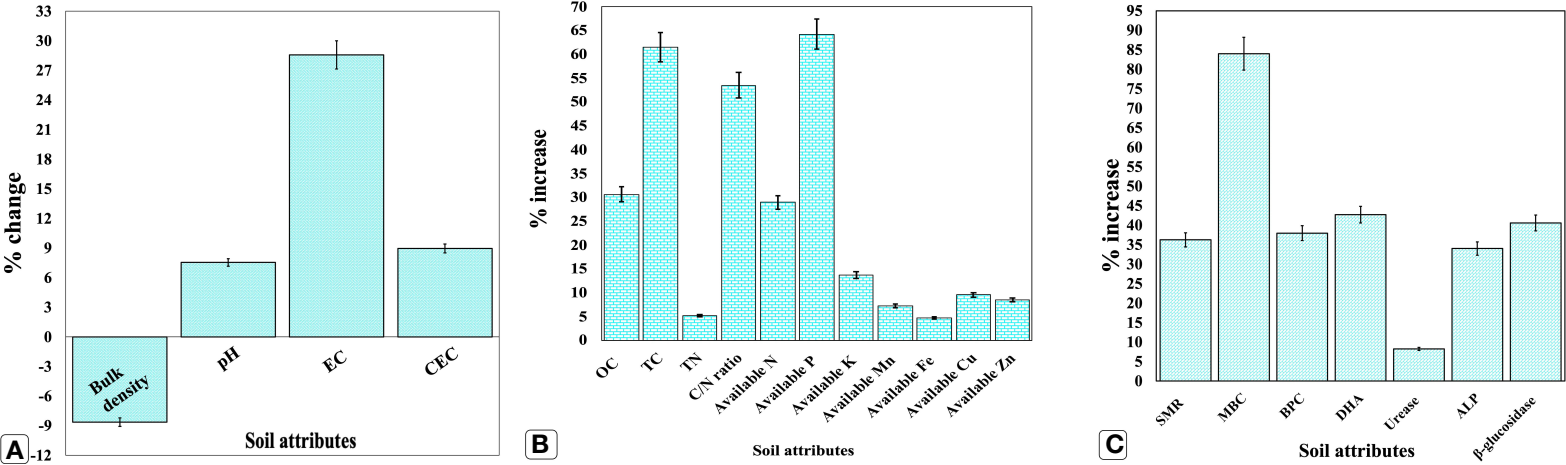
Effect of organic manure, biofertilizer, and seaweed extract on soil health indicators after two consecutive years. **(A)** changes in soil edaphic conditions; **(B)** increases in nutrient availability; **(C)** Increase in biological and enzymatic conditions. Results are the means of three replications (*n = 3*) ± SD. OC, organic carbon (%); TC, total carbon (g kg^−1^); TN, total nitrogen (g kg^−1^); C/N ratio: ratio of carbon to nitrogen; N, available nitrogen (kg ha^−1^), P, available phosphorus (kg ha^−1^); K, available potassium (kg ha^−1^); Mn, available manganese (mg kg^−1^); Fe, available iron (mg kg^−1^); Cu, available copper (mg kg^−1^); Zn, available zinc (mg kg^−1^); SMR, soil microbial respiration (CO_2_ g^−1^ dry soil day^−1^); MBC, soil microbial biomass carbon (mg C 100 g^−1^ dry soil); BPC, bacterial population count (× 10^7^ CFU g^−1^ soil); DHA, dehydrogenase activity (μg TPF g^−1^ dry soil h^−1^); Urease: urease activity (μg NH4^+^-N g^−1^ dry soil h^−1^); ALP, alkaline phosphatase activity (μg PNP g^−1^ dry soil h^−1^); β-glucosidase, β-glucosidase activity (μg PNG g^−1^ dry soil h^−1^); TPF, triphenyl formazan; PNP, p-nitrophenyl phosphate; PNG, p-nitrophenyl β-glucoside.

A remarkable amelioration in soil health contributing attributes was observed after the end of the two-year experiment as compared with the initial values (**Figures 2A–C**; **Supplementary Table S1**). Interestingly, a modest shift in soil pH from 5.3 to 5.7, a slight increase in cation exchange capacity (CEC), and electrical conductivity (EC) by 8.5 and 28.57%, respectively, were observed, indicating the positive effects of using organic biostimulants. The soil bulk density was reduced from 1.39 to 1.27 Mg cm^−3^. Conversely, no change in soil texture was observed, while OC was increased from 0.49 to 0.64%. In terms of soil nutrient availability, available macro and micronutrients *viz*., N, P, K, Mn, Fe, Cu, and Zn were increased by 28.90, 64.20, 13.62, 7.22, 4.63, 9.52, and 8.47%, respectively, compared with the initial values. Likewise, SMR, MBC, DHA, urease, ALP, and β-glucosidase activities were enhanced by 36.23, 83.98, 42.69, 8.24, 34.00, and 40.31%, respectively.

### Growth attributes

Significantly higher plant height (36.42, 70.69 cm) and primary branches (5.96, 10.22) at 50, and 100 DAT, respectively, were recorded during the second year compared with the first (**Table 3**). Among the vermicompost levels, significantly higher plant height (36.46, 69.69 cm) and the branches (6.77, 10.87) were recorded with the highest dose (8 Mg ha^−1^) when compared with moderate (4 Mg ha^−1^) and control (0 Mg ha^−1^) at 50 and 100 DAT, respectively. Likewise, biofertilizer significantly benefited all treated plants, leading to a 7.21 11.19% enhancement in plant height and branching by 39.96 24.32% at 50 and 100 DAT, respectively. On the contrary, foliar application of liquid seaweed extract at 7 ml L^−1^ showed a significant effect on plant height and branches at 100 DAT only.

**Table 3:** Effect of cropping year, organic manure, biofertilizer, and seaweed extract on growth attributes in *O. basilicum* at different growing intervals 50 DAT, and at harvest (100 DAT).

Treatments	Plant height (cm)			Primary branches plant^−1^			Plant spread N–S (cm)			Plant spread E–W (cm)	
	50 DAT	100 DAT		50 DAT	100 DAT		50 DAT	100 DAT		50 DAT	100 DAT
Cropping year											
2020	31.73^b^	54.19^b^		4.79^b^	8.04^b^		12.27^b^	25.39^b^		13.68^b^	25.10^b^
2021	36.42^a^	70.69^a^		5.96^a^	10.22^a^		14.36^a^	40.42^a^		18.18^a^	43.06^a^
SEm	0.39	0.33		0.08	0.16		0.38	0.38		0.57	0.23
LSD (*P = 0.05*)	1.10	0.94		0.23	0.47		1.08	1.09		1.63	0.67
Vermicompost											
V−	32.21^bc^	56.29^c^		4.08^c^	7.29^c^		11.05	27.08^c^		13.24^c^	29.17^c^
V4	33.54^b^	61.35^b^		5.28^b^	9.24^b^		13.54	31.84^b^		15.95^b^	32.90^b^
V8	36.46^a^	69.69^a^		6.77^a^	10.87^a^		15.35	39.80^a^		18.59^a^	40.17^a^
SEm	0.47	0.40		0.10	0.20		0.46	0.47		0.70	0.29
LSD (*P = 0.05*)	1.35	1.15		0.28	0.57		NS	1.32		1.99	0.81
Biofertilizer											
B−	32.89^b^	59.13^b^		4.56^b^	8.14^b^		12.45^b^	29.98^b^		14.52^b^	31.73^b^
B+	35.26^a^	65.75^a^		6.20^a^	10.12^a^		14.17^a^	35.84^a^		17.33^a^	36.43^a^
SEm	0.39	0.33		0.08	0.16		0.38	0.38		0.57	0.23
LSD (*P = 0.05*)	1.10	0.94		0.23	0.47		1.08	1.09		1.63	0.67
Seaweed extract											
S−	33.74	60.91^b^		5.03^b^	8.47^b^		12.97	31.31^b^		15.61^ab^	33.07^b^
S+	34.40	63.98^a^		5.72^a^	9.79^a^		13.66	34.50^a^		16.25^a^	35.09^a^
SEm	0.39	0.33		0.08	0.16		1.63	0.38		0.57	0.23
LSD (*P = 0.05*)	NS	0.94		0.23	0.47		NS	1.09		1.63	0.67

Results are the mean of three replications *(n = 3)* of two years pooled data, different lowercase letters in the same column of each treatment are significantly different at *(P = 0.05)*. DAT, days after transplantation; N–S, plant spread towards north to south direction; E–S, plant spread towards east to west direction; SEm, standard error of mean; LSD, least significant difference *(P = 0.05)*; NS, non-significant; V−, unfertilized control; V4, vermicompost at 4 Mg ha**^−^** ^1^; V8, vermicompost at 8 Mg h**^−^** ^1^; B, biofertilizer; S, seaweed extract at 7 ml L**^−^** ^1^; (−), without factor; (+): with factor.

### Leaf photosynthetic pigments and gaseous exchange

**Figure 3:**
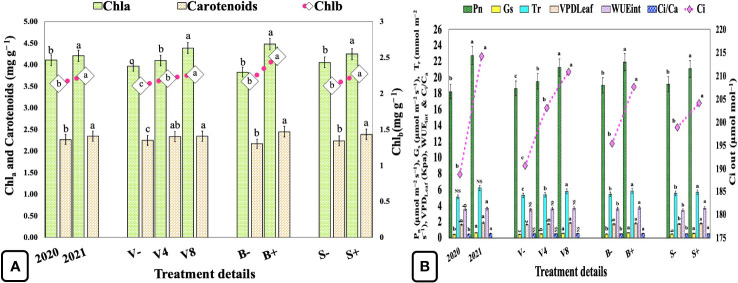
Effect of cropping year, organic manure, biofertilizer, and seaweed extract on leaf photosynthetic pigments and gaseous exchange in sweet basil. **(A)** Effect on photosynthetic pigments **(B)** Effect on photosynthetic characteristics. Data are pooled over two years, and results are represented as the means of three replications (*n=3*) ± SE, bars with different letters are significantly different at *P = 0.05*. NS, non-significant; V−, unfertilized control (no added manure); V4, vermicompost at 4 Mg ha^−1^; V8, vermicompost at 8 Mg ha^−1^; B−, uninoculated; B+, inoculated; S−, without seaweed extract; S+, foliar spray of seaweed extract at 7 ml L^−1^; Chl_a_, chlorophyll a; Chl_b_, chlorophyll b; P_n_, net photosynthetic rate; G_s_, stomatal conductance; C_i_, CO_2_ mole fraction in the leaf intercellular air spaces; T_r_, transpiration rate; VPD_Leaf_, leaf vapor pressure deficit; WUE_int_, intrinsic water use efficiency; C_i_/C_a_, ratio of intracellular CO_2_ to ambient CO_2_.

Concentrations of photosynthetic pigments Chl_a_, Chl_b_, and carotenoids were significantly enhanced by 2.43, 5.14, and 3.52%, respectively, in the second year when compared with the first (**Figure 3A**; **Supplementary Table S2**). Vermicompost at 4 and 8 Mg ha^−1^ significantly increased the Chl_a_ by 3.27, 10.57%, Chl_b_ by 4.74, 7.58%, and carotenoids by 4.00, 4.44%, respectively as compared with the unfertilized control (0 Mg ha^−1^). Similarly, biofertilizer and seaweed extracts have also significantly increased Chl_a_, Chl_b_, and carotenoids by 16.97, 16.12, 12.90%, and 4.68, 8.06, and 6.69%, respectively. The moderate and highest vermicompost dosages also significantly influenced the rate of photosynthesis by 4.56 and 13.96%, respectively, when compared with the unfertilized control. On the contrary, biofertilizer and seaweed extract significantly enhanced both P_n_ and WUE_int_ by 15.09, 5.90%, and 4.46, 7.58%, respectively (**Figure 3B**; **Supplementary Table S2**).

### Yield attributes and biomass yield

**Figure 4:**
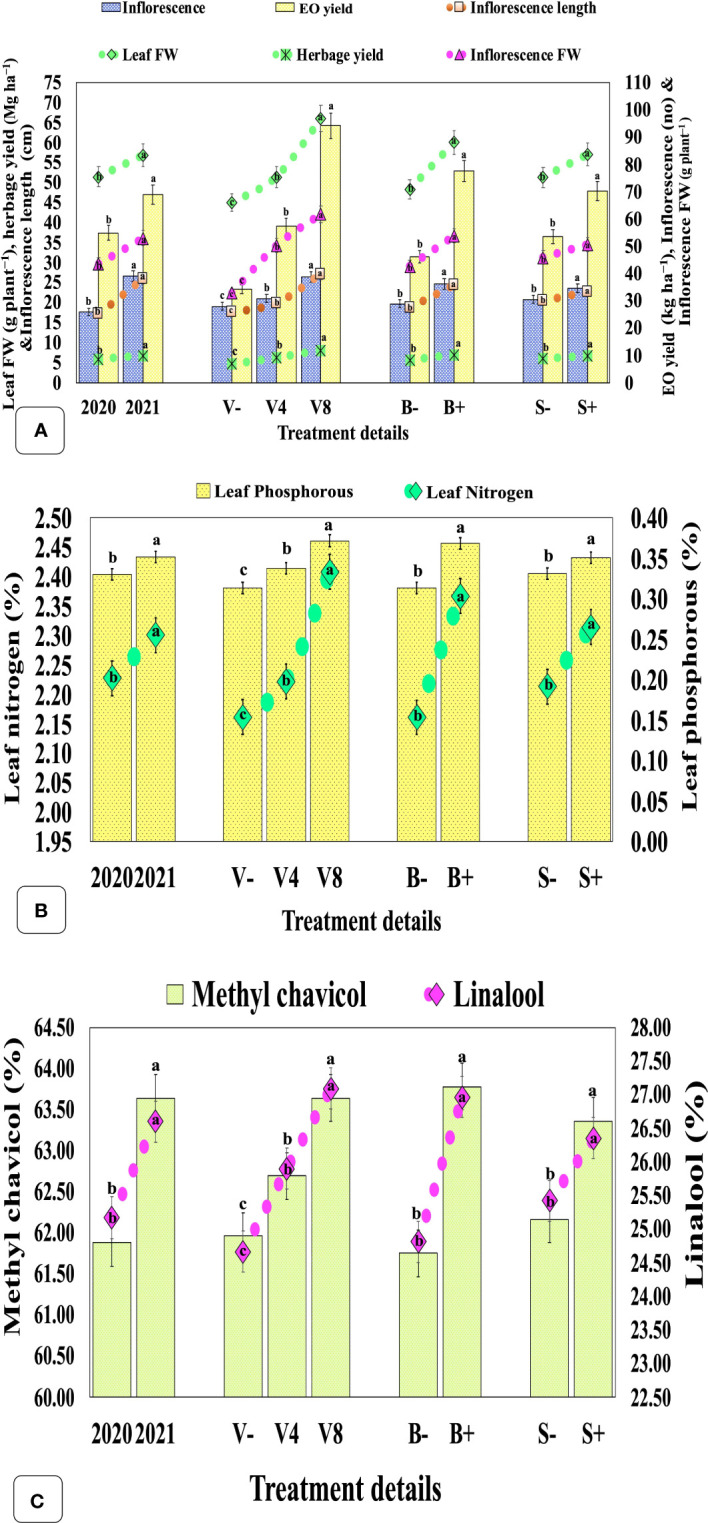
Effect of cropping year, organic manure, biofertilizer, and seaweed extract on yield, leaf mineral content, and secondary metabolites in sweet basil. **(A)** effect on yield, and associated attributes; **(B)** effect on leaf nitrogen and phosphorous contents; **(C)** effect on EO, methyl chavicol, linalool content. Data are pooled over two years, and results are represented as the means of three replications (*n = 3*) ± SE, bars with different letters are significantly different at *P = 0.05*. V−, unfertilized control (no added manure); V4, vermicompost at 4 Mg ha^−1^; V8, vermicompost at 8 Mg ha^−1^; B−, uninoculated; B+, inoculated; S−, without seaweed extract; S+, foliar spray of seaweed extract at 7 ml L^−1^; EO, essential oil; FW, fresh weight.

The herbage yield (leaf + inflorescence) was significantly higher (6.86 Mg ha^−1^) in the second year in contrast with the first year (6.01 Mg ha^−1^) (**Figure 4A**; **Supplementary Table S3**). The vermicompost amendment with the highest and modest dose significantly increased biomass yield by 69.85 and 31.60%, respectively, compared with the unfertilized control. Similarly, biofertilizer and seaweed extract significantly augmented herbage yield by 49.37 and 24.75%, respectively. A reverse trend was observed in the leaf+inflorescence/stem ratio for the ascending dose of vermicompost. Conversely, only biofertilizer significantly improved the ratio by 7.95%, whilst the foliar application of seaweed extract had no significant effect.

### Mineral concentration (N and P%) in leaves

Leaf N and P contents were significantly boosted by 3.29 and 6.46%, respectively, in the second year as compared with the first. The highest and moderate doses of vermicompost have significantly enhanced N and P concentrations by 11.41, 18.67%, and 2.80, 7.82%, respectively when compared with the unfertilized control. Similarly, seaweed extract and biofertilizer also significantly increased N and P contents by 4.56, 5.84%, and 9.53, 17.66%, respectively (**Figure 4B**).

### Essential oil percentage, composition, and yield

The EO content and yield were significantly higher in the second year (1.05%, 69.03 kg ha^−1^) when compared with the first (0.97%, 55.03 kg ha^−1^), respectively (**Figure 4C**; **Supplementary Table S3**). Among factors, a significantly higher EO concentration (1.03%) was observed in plants treated with 8 Mg ha^−1^ vermicompost, compared with control (0 Mg ha^−1^) but remained statistically at par with the moderate dose (4 Mg ha^−1^). Likewise, biofertilization and foliar application of seaweed extract also significantly affected EO content and yield by 45.35, 67.95%, and 18.48, 31.33%, respectively. The major constituents of EO, methyl chavicol and linalool, significantly increased by 2.84 and 5.68%, respectively, in the second year as compared with the first year (**Figure 4C**; **Supplementary Table S4**). The highest and moderate rates of vermicompost also significantly increased the concentration of methyl chavicol and linalool by 2.71, 9.85%, and 1.18, 5.03%, respectively, when compared with the unfertilized control. Similarly, the biofertilization and seaweed extract also enhanced their concentrations by 3.29, 8.67%, and 1.93, 3.66%, respectively.

### Correlations of traits and interaction effect of biostimulants (V × B × S)

**Figure 5:**
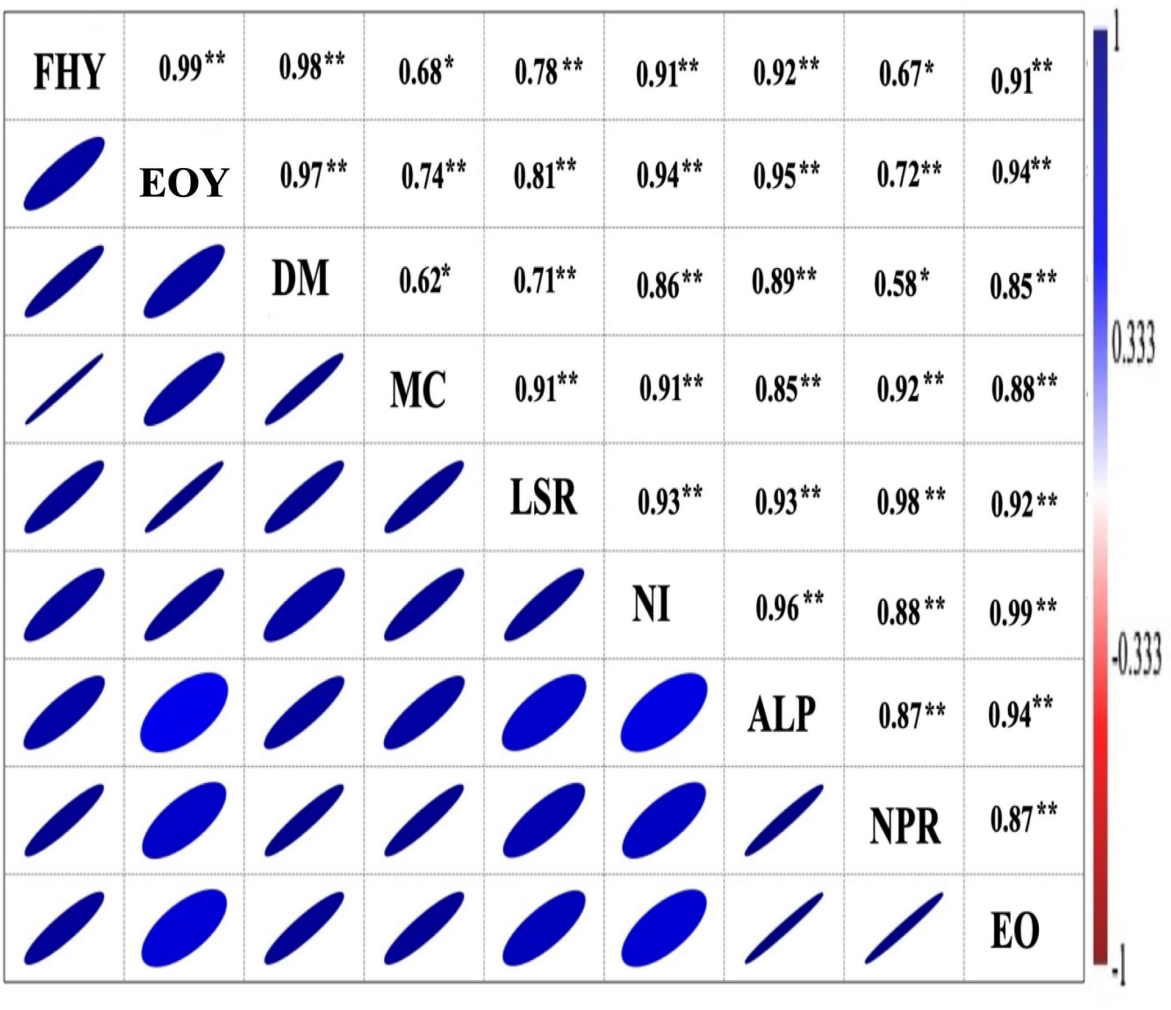
Correlation analysis between photosynthesis, yield parameters, and sweet basil’s marker compound. FHY, fresh herb yield; EOY, essential oil yield (kg ha^−1^); DM, dry matter plant^−1^; MC, methyl chavicol (%); LSR, leaf + inflorescence/stem ratio; NI, number of inflorescence plant^−1^; ALP, average length of panicle (cm); NPR, net photosynthetic rate (μmol m^−2^ s^−1^); EO, essential oil (%). The mean values of three biological replicates of the corresponding treatments (12) were (N3:N:2:N:2 = 12) used, * and ** indicate the significant differences between corresponding values at *P = 0.05* and *P = 0.01*, respectively.

**Figure 6:**
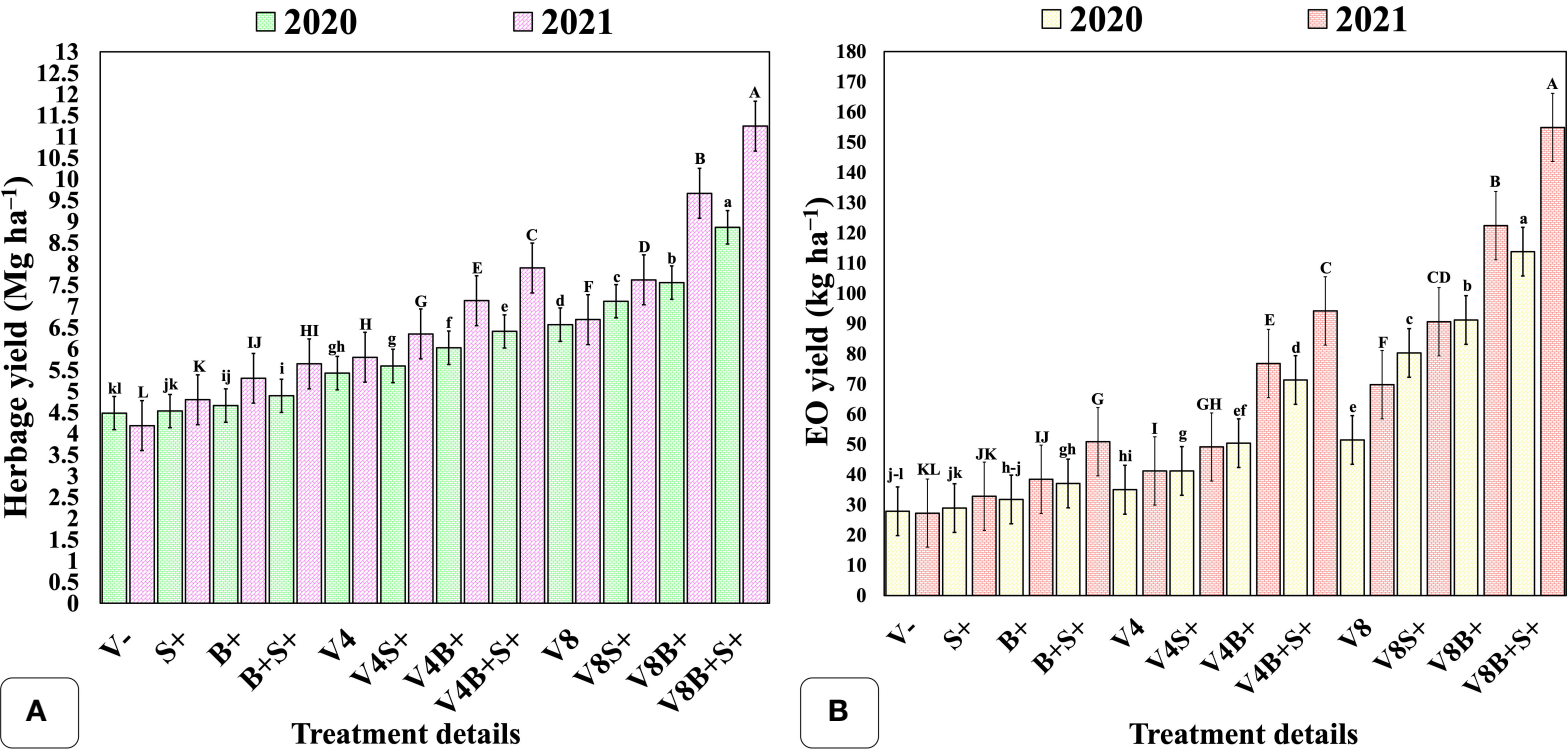
Interaction effect of organic manure, biofertilizer, and seaweed extract on yield components of sweet basil. **(A)** herbage yield (Mg ha^−1^); **(B)** EO yield (kg ha^−1^). Data are represented as the means of three replications (*n = 3*) ± SE, bars with different letters are significantly different at *P = 0.05*. V−, unfertilized control (no added manure); V, vermicompost; V4, vermicompost at 4 Mg ha^−1^; V8, vermicompost at 8 Mg ha^−1^; B, biofertilizer; S, seaweed extract; (−), without factor; (+), with factor. Other abbreviations are provided in **Table 1**.

**Figure 7:**
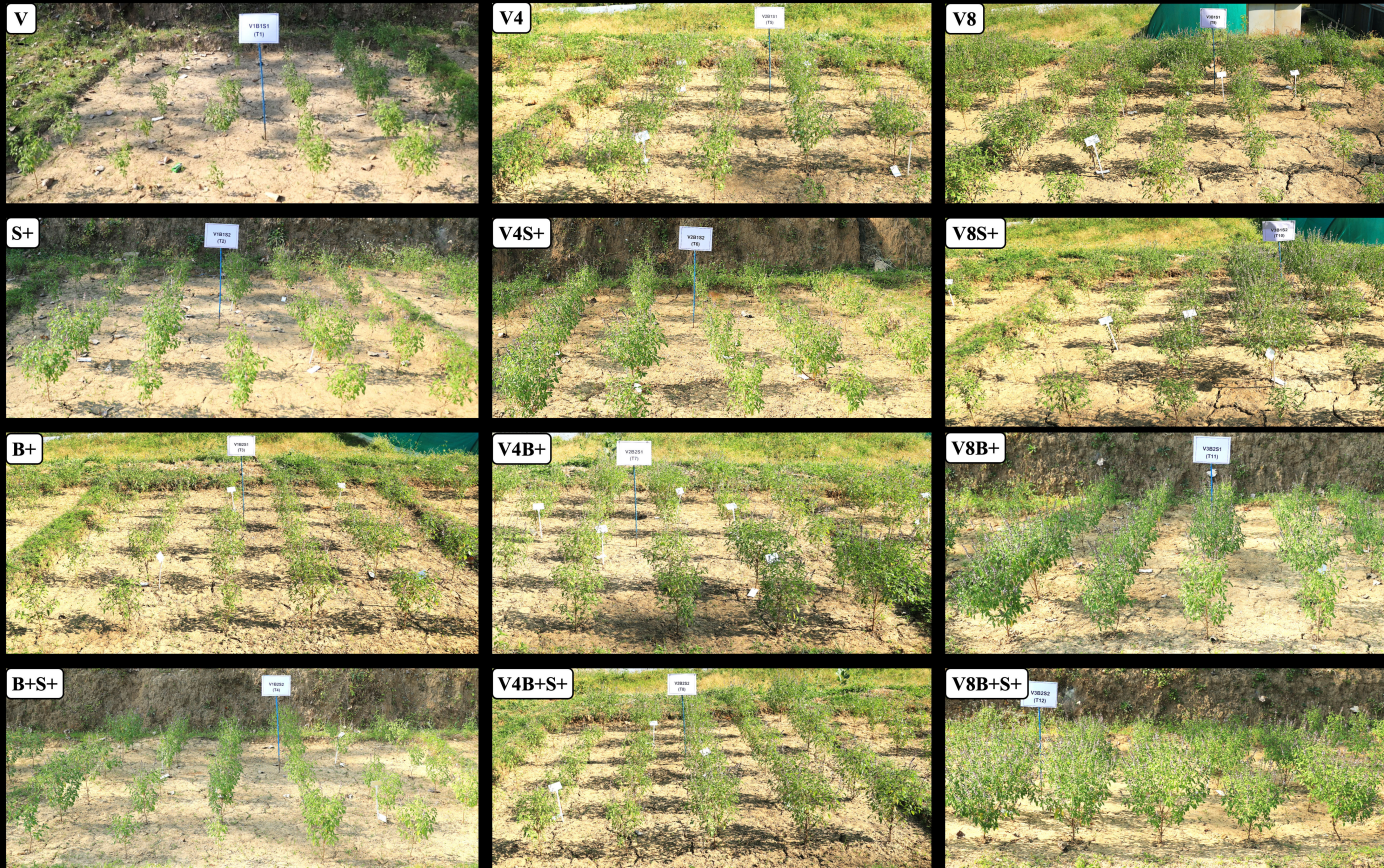
Treatment-wise representative experimental plots of *O. basilicum* in the Indian western Himalaya. V, vermicompost; V−: unfertilized control (no added manure); V4, vermicompost at 4 Mg ha^−1^; V8, vermicompost at 8 Mg ha^−1^; B, biofertilizer; S, seaweed extract; (−), without factor; (+), with factor. Other abbreviations are provided in **Table 1**.

The matrix of correlation (**Figure 5**) was significant (*P = 0.01*) and showed a positive correlation of EO yield with herb yield (r = 0.91), EO content (r = 0.94), dry matter content (r = 0.97), leaf + inflorescence/stem ratio (r = 0.81); number of inflorescence plants^−1^ (r = 0.94); average length of panicle (r = 0.95); net rate of photosynthesis (r = 0.72). Furthermore, the methyl chavicol content was also positively correlated at *P = 0.01* with all chosen traits, except for dry matter content, which was significant at *P = 0.05* (r = 0.62). Similarly, the herbage yield was positively correlated (*P = 0.01*) with all considered attributes, excluding the net photosynthetic rate, which was significant at *P = 0.05* (r = 0.67). Biomass and EO yield were both significantly influenced by the interaction effect of all three involved factors, *viz*., vermicompost (V), biofertilizer (B), and liquid seaweed extract (S) during both years (**Figures 6A, B**). In both years, the maximum herb and EO yield were recorded as 8.86, 11.25 Mg ha^−1^ and 113.78, 154.87 kg ha^−1^, respectively in T12 (V8B+S+) receiving congregate treatment of all three biostimulants (**Figure 7**).

## Discussion

Assuring sustainable production of salubrious crop products for environmental safety and socio-economic issues is not only a critical topic for agriculture, ecology, and the environment, but also pondered by farmers, researchers, policymakers, and stakeholders. However, farmers are accustomed to using a lot of energy, such as chemical fertilizers, to augment plant growth and productivity, which has increased CO_2_ concentration, temperature, GHG emissions, environmental pollution, and unintentionally deteriorated soil health, including crop product quality. Moreover, organic farming practices are the only way to keep the soil in good shape and increase agricultural production in the long term. However, the biggest disadvantage of organic farming is its lower and irregular output when compared with conventional farming. Consequently, rigorous research is needed to understand how biostimulants may be employed to alleviate nutrient limitations by boosting nutrient absorption, and ultimately cutting short the apparent yield gap between organic and traditional output. Furthermore, the sole application of biostimulants cannot meet the requirements for a viable alternative to mineral fertilizers. Therefore, the present investigation was performed to develop innovative and robust yet sustainable production technologies for the cleaner production of Indian sweet basil by using a combinatorial application of a diverse range of biostimulants under the rainfed conditions of Indian western Himalaya. The findings of the current investigation not only revealed the beneficial effects of used organic biostimulants on sweet basil growth, physiological performance, biomass, EO yield, and composition but also meliorated soil health.

### Soil health

Soil health is an intricate result of a complex interaction between the soil’s physical, biological, and chemical components. Agronomical measures like soil management can have a significant impact on these components (Zhang et al., 2020; Hu et al., 2021). Despite the important influence of organic biostimulant amendments on soil quality, only a few studies are available that incorporate all these three soil quality indicators. Therefore, in the current investigation, we tried to decipher the effect of biostimulants on all three soil components. In terms of soil physical properties, a modest increase in soil pH was noticed (**Figure 2A**; **Supplementary Table S1**), making it more favorable for soil processes like nutrient availability and microbial activity, as the pH range between 5.5 and 8.0 provides favorable optimal circumstances for almost all soil activities. The present results are in congruence with previous findings reporting an increase in soil pH towards neutrality by adding vermicompost and phosphate solubilizing bacteria (PSB) through organic compound creation during the decomposition of complex organic molecules and mineralization (Goswami et al., 2017). Additionally, the soil pH might have increased due to the presence of sodium ions (Na^+^) in seaweed extract (Lötze and Hoffman, 2016). In contrast, soil bulk density declined, possibly due to the addition of organic carbon (OC) (**Figure 2B**), which might have increased the pore space and thereby the soil aggregation (Gourav et al., 2019). Interestingly, a slight increase in soil EC and CEC was also noticed, which is accountable for the large negative charge of biological materials accumulated as of biostimulant amendments; being critical for nutrient retention and its availability to plants (Choudhary et al., 2021).

Among soil biological properties, SMR and MBC denote the estimation of CO_2_ liberated by soil microflora in the respiration process and the extent of carbon fixed in soil microorganisms’ cell structures, respectively. Both are connoted by the soil microbial population and their activity (Parastesh et al., 2019). A significant enhancement in SMR and MBC can be attributed to the increased BPC (**Figure 2C**), as organic amendments can act as substrates, supply soluble nutrients to soil microflora, and eventually augment the relative abundance of the soil bacterial population (Sarma et al., 2018). Accounting for the soil enzymatic traits, the increased DHA could be attributed to enhanced SMR and BPC (**Figure 2C**) by organic additives, resulting in improved soil metabolic activities (Hamdi et al., 2019). Likewise, ALP catalyzes the hydrolysis of insoluble organic phosphomonoester to inorganic P, releasing orthophosphates that could be easily digested by plants and soil microbes. Moreover, it was already discovered that vermicompost and bioinoculants could boost soil ALP activity due to increased inorganic soluble phosphates (Hussain et al., 2016). Similarly, the mineralization and mobilization of NPK by biofertilizers might have augmented the soil-accessible NPK (**Figure 2B**; **Supplementary Table S1**), as microbial biostimulants actively participate in the mineralization of fixed nutrients into accessible ones by accelerating the rate of biogeochemical cycling. Moreover, vermicompost contains HSs, C, P, and N, and a diverse range of PGPRs (Pathma and Sakthivel, 2013), which could also be the source of the observed increase in soil-accessible NPK after the end of the two-year experiment. Furthermore, organic materials such as vermicompost, seaweed extracts, and bioinoculants have demonstrated a significant increase in soil health in some earlier investigations (Verma et al., 2016; Gupta et al., 2019; Trivedi et al., 2022) due to improved microbial community structure and soil enzymatic activities, which are in coherence with the current study.

### Growth and yield

All growth-contributing attributes were significantly higher in the second year (**Table 3**) as compared with the first. This might be due to biostimulant amendments for two consecutive years leading to a significant improvement in soil health (**Figures 2A–C**; **Supplementary Table S1**), as they contain a considerable amount of macro and micronutrients, including organic matter (**Table 2**). Furthermore, despite receiving ~16.83% higher rainfall in the second year (**Figure 1B**), the crop yield was consistent or even far better than the first year. This might be attributable to the stress alleviation properties of biostimulants delivering better resilience to crops to confront various environmental stressors. The PGPR strains used as biofertilizers in the current investigation have the potential to demonstrate ACCD activity (**Table 2**), which alleviates the stressors by preventing the formation of ethylene under waterlogging conditions (Barnawal et al., 2012). Also, seaweed extracts are well-documented as stress alleviators because of the osmolytic properties of glycine, betaine, and choline chloride in their composition (Singh et al., 2016b). The biofertilizer can stimulate plant growth through biological nitrogen fixation (BNF), regulation of PGRs and expression of nitrate transporter genes (*NRT1.1*, *NRT2*, and *NAR2.2*), solubilization of insoluble compounds like calcium di, tri-phosphates, and siderophore production (Van Gerrewey et al., 2020; Song et al., 2021). Interestingly, a recent study demonstrated that CK, a PGR released by beneficial microbes, augments crop productivity, as microbial-produced CKs could promote plant metabolism, leading to increased vigor and also recruit disease-protective microbiome, finally increasing the overall benefit to the host plant (Gupta et al., 2022). Moreover, the combined application of vermicompost and biofertilizer might have enhanced nutrient acquisition, plant growth-promoting enzymes, and soil microbial diversity and ultimately increased the overall productivity of the crop (Misra et al., 2019). Current findings are consistent with some former investigations, reporting a significant increase in crop biomass using organic manure in *O. basilicum* (Cabanillas et al., 2013) and biofertilizer in *Mentha arvensis* L. (Singh et al., 2019).

Additionally, seaweed extracts can also boost crop growth. Still, the exact process remains elusive; it is assumed that the plausible activity of seaweed extracts is due to the presence of macro/micronutrients, PGRs, betaines, and phenolic compounds in their composition (**Table 2**). In a prior study, foliar spraying with *K. alvarezii* and *Gracilaria edulis* seaweed saps significantly increased the yield and quality of maize. This improvement was attributed to higher photosynthetic capacity, higher net assimilation rate, and the impact of various PGRs as well as other substances present in these seaweed saps (Singh et al., 2016b). Similarly, a recent root transcriptome analysis revealed that the soil drench with seaweed extract (*K. alverazii*) in *Zea mays* L. upregulates genes related to root growth, PGR signaling, stress responses, plant nutrition, and transport, including DNA repair under drought conditions (Kumar et al., 2020). Sequentially, a recent leaf transcriptomic study revealed that the foliar spray of seaweed extract (*K. alverazii*) induces the photosynthesis and starch biosynthesis associated genes in *Z. mays*, leading to higher biomass production (Trivedi et al., 2021). Owing to these previously reported observations, it was speculated that in the present study, sweet basil might have experienced a similar effect like enhanced photosynthetic pigments (**Figure 3A**), rate of photosynthesis (P_n_) (**Figure 3B**), and eventually produced higher crop biomass after foliar application with liquid seaweed extract.

### Leaf photosynthetic pigments, mineral nutrients, and gas exchange

All biostimulants significantly enhanced the concentrations of photosynthetic pigments as compared with the unfertilized control (**Figure 3A**). Similar findings were reported by others in *Amaranthus hybridus* L. after foliar application with some natural biostimulants like smoke-water, karrikinolide, vermicompost leachate, Kelpak^®^, and eckol (Ngoroyemoto et al., 2019) and also in *Vigna unguiculata* (L.) Walp after treatment with seaweed extract (Kelpak^®^) and vermicompost leachate under drought stress (Voko et al., 2022). Likewise, biofertilizer application can also increase the number of photosynthetic pigments as there is a well-established link between total chlorophyll content and increased iron acquisition facilitated by PGPRs through iron-containing enzymes and siderophores (Eshaghi Gorgi et al., 2021). Furthermore, the higher leaf N concentrations (**Figure 4B**) might have enhanced the content of chlorophyll (**Figure 3A**), as thylakoid N is directly proportional to the chlorophyll content. Additionally, manganese (Mn) plays a pivotal role in plant metabolisms like enzyme activation, nitrate reduction, amino acid, chlorophyll, and protein synthesis, including photosynthesis and phytohormone regulation (Ghannadnia et al., 2014). Therefore, the increased concentration of photosynthetic pigments can also be ascribed to the improved status of Mn in soil (**Figure 2B**; **Supplementary Table S1**).

Similarly, biofertilizers can also influence the photosynthetic process by altering the auxin pool, which aids the plant’s root system and allows better acquisition of mineral nutrients, leading to the accumulation of higher photosynthetic pigments and gaseous exchange (Liu et al., 2019; Samani et al., 2019). However, the association between photosynthesis and PGPRs is commonly given only in an indirect way, albeit, in the current investigation, a direct and pronounced favorable effect of biofertilizers on photosynthesis-associated characteristics was observed (**Figure 3B**). As a result, the quinone acceptors (Q_a_) are substantially oxidized and their excitation energy is used in electron transport, resulting in the increased synthesis of energy molecules (ATP and NADPH). These energy molecules are further used for carbon assimilation during the C_3_ cycle and eventually increase crop biomass (Rozpądek et al., 2015). Additionally, an increase in N and P content in leaves treated with seaweed extract might be due to the considerable increase in transcript abundance of genes related to N and P assimilation in leaves, including increased nitrate and phosphate transporters and nitrate reductase activity in roots, as earlier reported in *Z. mays* (Trivedi et al., 2018a; Trivedi et al., 2021). Furthermore, CO_2_ produced during SMR can also enhance the rate of photosynthesis (P_n_). Therefore, the increased SMR (**Figure 2C**) can also be ascribed as a putative reason for the enhanced net photosynthetic activity observed in the current study.

### Essential oil content, yield and composition

The observed variabilities in EO content and composition as of the exogenous application of biostimulants in the current investigation are in agreement with earlier studies that agronomic practices (Choudhary and Kumar, 2019; Rathore and Kumar, 2021), genomic makeup, environmental factors, soil edaphic conditions, and ontology (Esmaeili et al., 2018) all can influence the quantity and quality of EO in MAPs. Furthermore, plants require micronutrients, especially Zn and Cu, for cell division, photosynthesis, electron transport chain (ETC), chlorophyll, protein, and auxin synthesis. Additionally, these act as metal constituents of several enzymes and regulatory cofactors involved in saccharide metabolism (Tavallali et al., 2018). The higher availability of micronutrients in soil (**Figure 2B**; **Supplementary Table S1**) could be attributed to the observed increased EO content under organic biostimulant amendments (**Figure 4C**). Similarly, multiple studies have already reported that the organic amendment and bioinoculant can increase EO in various MAP_s_ like *Dracocephalum moldavica* L. (Vafadar-Yengeje et al., 2019), *D. kotschyi* Boiss. (Fallah et al., 2020), and *O. basilicum* (Yilmaz and Karik, 2022). Furthermore, both N and P are critical for the production and activation of enzymes involved in various biochemical reactions. Therefore, the increased N and P contents in the current investigation (**Figure 4B**) due to organic biostimulants might have enhanced EO content in sweet basil (**Figure 4C**; **Supplementary Table S4**); as the formation of EO-carrying cells, channels, glandular trichomes, and secretory ducts are all aided by these two moieties (Rostaei et al., 2018). Moreover, N may boost electron transport rates (ETR), RuBisCO activity, and photosynthesis, which provides ATP and carbon substrate for isoprene synthesis and ultimately increases the accumulation of EO (Machiani et al., 2018). Alike, P being a component of EO precursors (isopentyl diphosphate and dimethylallyl diphosphate) can also enhance EO production (Dehsheikh et al., 2020). The observed increase in EO yield reported in the current investigation might be attributed to the increased number of glandular trichomes and antioxidants, as some posterior studies have already claimed these factors are responsible for the increase in EO yield after combined treatment of plant beneficial microbes in MAPs like sweet basil (Gupta and Pandey, 2015) and *Pelargonium graveolens* L’Hér (Gupta et al., 2016).

The biosynthetic pathways of EO components are well-recognized to be extensive and complex. Despite extensive research that has been conducted to elucidate the plausible mechanism of biosynthesis, the exact relation between biosynthetic routes of various constituents remains elusive. Nonetheless, it is well known that terpenoid biosynthesis is dependent on primary metabolisms like photosynthesis and oxidative pathways for carbon and energy supply, and thus the plants inoculated with biofertilizer can produce more primary metabolites through boosting photosynthesis and other metabolic activities, which can eventually enhance the secondary metabolite accumulation (Banchio et al., 2010). Furthermore, the activation/inactivation of enzymes involved in EO biosynthetic pathways like mevalonate (MVA), methylerythritol phosphate (MEP), and shikimic acid could also cause alterations in constituents of EO (Gupta et al., 2020). Interestingly, in the current investigation, methyl chavicol and linalool content were increased with all three amended biostimulants (**Figure 4C**). On the contrary, only biofertilizer application has exorbitantly boosted α-Bergamotene (**Supplementary Table S4**), which has the highest binding affinity for Angiotensin-Converting Enzyme 2 (ACE2) protein among all available antiviral medicines for SARS CoV2 (Duru et al., 2021). The plausible mechanism behind this could be the alterations in expression patterns of the genes involved in the putative biosynthetic pathway of terpenoid biosynthesis. Moreover, not only the application of live cultures but also their metabolites can alter the secondary metabolite pathways in MAPs; as a prior study demonstrated an increase in withanolide A content along with growth in *Withania somnifera* (L.) Dunal after inoculation with cell pellets and metabolites of PGPRs like *B. subtilis* and *Streptomyces* sp. at 10 ml plant^−1^ pot^−1^ under a poly greenhouse condition (Singh et al., 2016a). Sequentially, another study also showed ~1.5-fold augmentation in bacoside A production in *Bacopa monnieri* (L.) Pennell inoculated with chitinolytic microbes as of alteration in the biosynthetic pathway of bacoside A (Gupta et al., 2017). In addition, it was hypothesized that the observed increase in EO and secondary metabolite production might be linked to elicitors (PGRs and other biologically active chemicals) produced by biostimulants, which eventually stimulated the secondary metabolite synthesis *via* an induced systemic resistance (ISR) mechanism, leading to activation of the host plant’s chemical defense cascades like jasmonic acid (JA) and ethylene (ETH), as many studies have successfully demonstrated the occurrence of ISR in MAPs after biostimulation (Egamberdieva et al., 2015; Chamkhi et al., 2021).

### Interaction effect of biostimulants

The current investigation demonstrated a synergistic effect of used biostimulants and reported the maximum herb and EO yields were observed when the crop was treated with the congregate application of all three biostimulants (**Figures 6A, B**). These findings are in congruence with some posterior studies demonstrating a synergistic/additive effect of the combined application of a diverse array of biostimulants for augmenting plant growth, production, and even biochemical composition in crops like lettuce (Rouphael et al., 2017; Rasouli et al., 2022), green amaranth (Ngoroyemoto et al., 2020), onion (Gupta et al., 2021a), and wheat (Najafi Vafa et al., 2022). Moreover, the observed beneficial effects of the combinatory use of biostimulants in the current study might be attributable to the increased photosynthetic pigments, physiological performance, and enhanced N and P contents in the leaves of *O. basilicum* (**Figures 3A–C**). Additionally, the plausible action mechanism of enhanced crop performance might be associated with the improved soil health status (**Figures 2A–C**) coupled with enhanced bacterial abundance and nutrient use efficiency (**Figure 4B**) driven by the synergistic action of vermicompost, biofertilizer, and seaweed extract. Concurrent observations were reported in a previous investigation in which a combined application of *Trichoderma*-based biostimulant at 50 g L^−1^ and seaweed extract at 2 g L^−1^ derived from *Ascophyllum nodosum* interacted synergistically and augmented the growth, nutritional and functional quality of organically grown tomatoes. In addition, they also reported a significant increase in soil fertility by fostering the growth of rhizospheric microbial populations, thereby increasing nutrient use efficiency, plant growth, and levels of antioxidant enzymes (Sani et al., 2020).

### Elucidation of plausible bio-stimulatory mechanism

**Figure 8:**
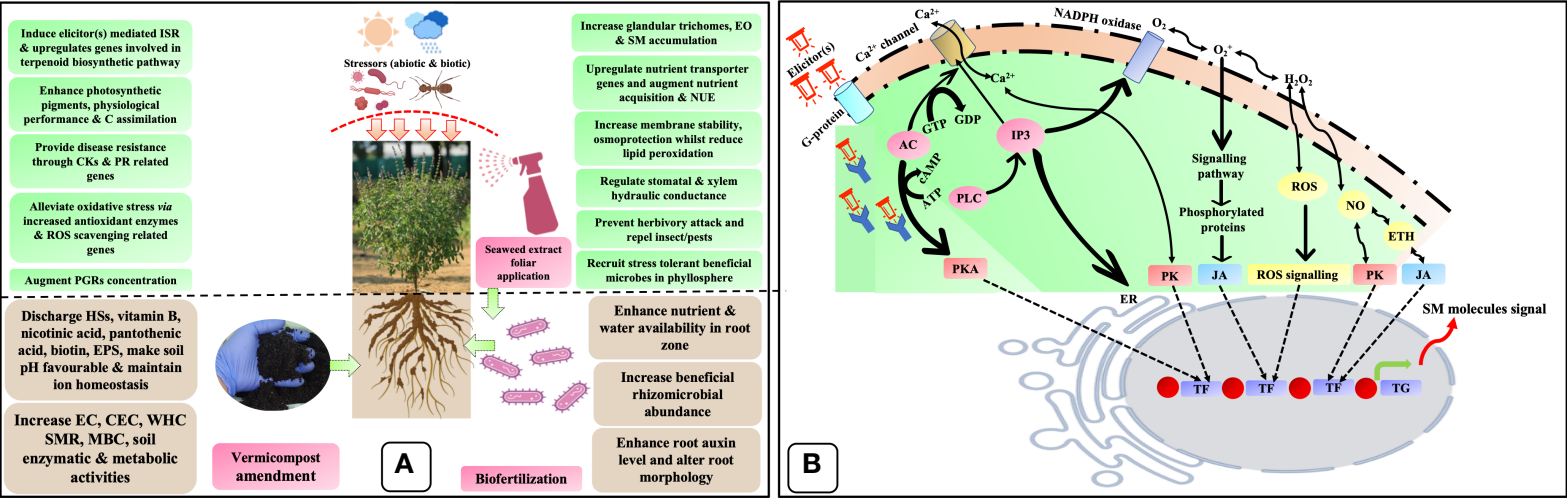
Hypothetical bio-stimulatory action mechanisms of biostimulants. **(A)** Plausible mode of action of biostimulants (vermicompost, biofertilizer, and seaweed extract) upon interaction with plants and their growing environment; **(B)** Plausible bio-stimulatory signaling pathways for elicitation of secondary metabolite synthesis. HSs, humic substances; EPS, exopolysaccharide; PGRs, plant growth regulators; CKs, cytokinins; PR, pathogenesis-related; C, carbon; ISR, induce systemic resistance; EO, essential oil; NUE, nutrient use efficiency; EC, electrical conductance; CEC, cation exchange capacity; WHC, water holding capacity; SMR, soil microbial respiration; MBC, microbial biomass carbon; G-protein, guanine nucleotide-binding proteins; AC, adenylate cyclase; IP3, inositol trisphosphate; ER, endoplasmic reticulum; NADPH oxidase, nicotinamide adenine dinucleotide phosphate oxidase; GTP, guanosine triphosphate; GDP, guanosine diphosphate; ATP, adenosine triphosphate; cAMP, cyclic adenosine monophosphate; PLC, phospholipase C; PKA, proteins kinase A; PK, proteins kinase; H_2_O_2_, hydrogen peroxide; OH, hydroxy free radical; ^1^O_2_, singlet oxygen; ROS, reactive oxygen species; NO, nitric oxide; ETH, ethylene; TFs, transcriptional factors; TG, target gene; SMs, secondary metabolites.

The plausible implication of biostimulants on several agronomical, biochemical, physiological, and molecular processes governing plant growth, productivity, quality, and resilience was adopted by Sani and Yong (2021) and illustrated in **Figure 8A**. The bio-stimulatory effect of biofertilizer could be explained by several mechanisms: i) improving soil pH, EC, CEC, WHC, nutrient availability, plant uptake, and assimilation; (ii) modification of root architecture; (iii) enhancing the physiological performance of plants; (iv) bolstering the antioxidant defense system; (v) PGR production and regulation; (vi) Upregulation of nutrient assimilation and transporters genes such as *AMT*, *NRT1.1*, *NRT2*, *NAR2.2*, *Pht1*, *PT2-1, DULTR4.2*, and *SULTR4.1*; and (vii) alteration of rhizomicrobiome through the production of enzymes and organic compounds. Likewise, the seaweed extract can augment plant growth and development through hormonal homeostasis, upregulation of nutrient transporter genes, stimulation of photosynthesis, and increased stress tolerance due to antioxidant stimulation and decreased lipid peroxidation, including reactive oxygen species (ROS) levels (Trivedi et al., 2018b). Additionally, the presence of polysaccharides, betaines, polyamines, phenolic compounds, and phytohormones in seaweed extracts may influence several signaling pathways and gene expression, therefore, imparting a positive effect on plants. Furthermore, seaweed extract induces bacteria-plant signaling *via* stimulatory effects, activates bacterial genes (*nodC*), and recruits beneficial microflora in the phyllo and rhizosphere. Likewise, vermicompost’s bio-stimulatory action is due to the presence of HSs, PGRs, PGPRs, and other advantageous substances in its composition. The biostimulatory activity of HSs has been attributed to numerous mechanisms (i) increasing WHC and CEC, neutralizing soil pH, and meliorating the soil structure; (ii) improving P solubility by preventing its precipitation and also preventing leaching, thereby augmenting the availability of nutrients, (iii) act like auxin and improve lateral root induction thereby, trigger the plasma membrane H^+-^ATPase activity, and (iv) stimulates nitrate assimilation *via* upregulation of the target genes/enzymes. Additionally, HSs can decrease hydrogen peroxide (H_2_O_2_) and lipid peroxidation, which consequently raises the proline concentration and recruits beneficial stress-resilient microbial communities in the rhizosphere (Xu et al., 2020).

Furthermore, it is well known that the plant G-proteins (guanine nucleotide-binding proteins) are associated with the response to biostimulants and other cellular processes related to growth, hormonal signaling, and defensive responses. The elicitor(s) produced by biostimulants serve as ligands and are identified by PRR (pattern-recognition receptors) to create a ligand–receptor complex. In this manner, a plant cell cognizes the signal produced by the elicitor(s) (Yakhin et al., 2017). Upon reception, the transduction signal produces many secondary messengers, *viz*., calcium ion (Ca^2+^), JA, salicylic acid (SA), nitric oxide (NO), and ROS, leading to enhanced secondary metabolite (SM) accumulation in MAPs (**Figure 8B**). When elicitor(s) bind to membrane receptors like plant G-protein, the transmembrane domain is activated and GTP is degraded to GDP *via* GTPase activity. This signaling event activates adenylate cyclase (AC) activity and converts adenosine triphosphate (ATP) to cyclic adenosine monophosphate (cAMP). As a result, phosphorylated protein kinase A (PKA) stimulates transcriptional factors (TFs) of the target gene (TG). Furthermore, inositol trisphosphate (IP3) is produced by phospholipase C (PLC) activity stimulated by G-protein activation. This secondary messenger increases intracellular Ca^2+^ by activating Ca^2+^ channel efflux and its binding to a particular receptor on the endoplasmic reticulum (ER). The augmented intracellular Ca^2+^ concentrations cause phosphorylation of certain proteins like proteins kinase (PK), which regulates gene expression by activating TFs. Additionally, activated PLC is also implicated in generating oxidative bursts by producing H_2_O_2_, hydroxy free radical (OH), singlet oxygen (^1^O_2_), and superoxide anion (O_2_) through swapping between O_2_ and H_2_O_2_ depending on NADPH oxidase and peroxidase activity in the plasma membrane. Consequently, H_2_O_2_ generation in the plasma membrane leads to stimulation of ER through increased cytosolic Ca^2+^ concentration. Furthermore, these molecules also trigger secondary messengers like JA, SA, NO, PK, ETH, and ROS that can modify the epigenetic pathways regulating gene expression. Likewise, various secondary messengers regulate the expression of genes by transforming the methylation state of DNA into a hypo-methylation state to permit access to TFs and RNA polymerases to the promoter region. Moreover, secondary messengers can also relax the structure of chromatin by shifting it to a euchromatin state, which also allows access of TFs and RNA polymerases to the promoter region. These epigenetic state transitions mediated by secondary messengers allow gene expression flexible in the presence of elicitor(s). In this manner, the plant cell genome incorporates elicitor(s) signals *via* epigenomic variations and thereby increases the production of secondary metabolites for its defense (Chamkhi et al., 2021).

## Conclusion

The pursuit of cleaner crop production is to improve nutrient availability and nutrient use efficiency while preserving soil health and producing quality crop products to slacken reliance on agrochemicals. In this regard, the current study unequivocally alludes that the congregate use of vermicompost, biofertilizer and seaweed extract can be practiced by MAP growers and stakeholders for the cleaner and environment-friendly production of Indian sweet basil EO. In essence, the current field experiment suggests that the combinatorial application of organic biostimulants having harmonizing properties not only significantly enhanced crop yield but also improved soil health. Based on two years of crop productivity, averred maximum EO (~135 kg ha^−1^) was recorded, when the crop was treated with a congregate dose of vermicompost (8 Mg ha^−1^), biofertilizer, and foliar application of liquid seaweed extract (7 ml L^−1^). This production technology can be considered inexpensive and environmentally sustainable, having low carbon footprints and no harmful field emissions. Therefore, it can be concluded that the combination of microbial and non-microbial biostimulant categories can act synergistically and maximize the soil health and quality production of sweet basil EO, particularly under the rainfed conditions of the Indian western Himalayas. Nevertheless, more rigorous field evaluations are required to evaluate and legitimize the interaction effects of the diverse range of biostimulants, especially on MAPs in a range of agroclimatic conditions. In addition, deciphering the combinative effects of biostimulants on functional rhizomicrobiomes would allow us to have a better comprehension of the soil–microbe–plant nexus. Moreover, the exact mechanism of biostimulation is not fully understood and further investigations are required to discern the fine-tuning between biostimulants and the host plant by evaluating the global changes in the abundance of mRNA transcripts using a comparative high-throughput RNA-seq approach.

## Data availability statement

The original contributions presented in the study are included in the article/**Supplementary Material**. Further inquiries can be directed to the corresponding author.

## Author contributions

YR: Conceptualization, methodology, investigation, formal analysis, data acquisition, and curation, writing—original draft, and writing—review and editing. NA: Methodology. AP: Investigation and supervision. RK: Funding acquisition, conceptualization, investigation and supervision, validation, and writing—review and editing. All authors contributed to the article and approved the submitted version.

## Funding

The financial grant from the Council of Scientific and Industrial Research, New Delhi, India, under the CSIR Aroma Mission Phase II (HCP-0007) is also acknowledged.

## Acknowledgments

The authors are grateful to the Director, CSIR-IHBT, Palampur, India, for providing the necessary facilities for study. This manuscript represents the CSIR-IHBT communication number 5082.

## Conflict of interest

The authors declare that the research was conducted in the absence of any commercial or financial relationships that could be construed as a potential conflict of interest.

## Publisher’s note

All claims expressed in this article are solely those of the authors and do not necessarily represent those of their affiliated organizations, or those of the publisher, the editors and the reviewers. Any product that may be evaluated in this article, or claim that may be made by its manufacturer, is not guaranteed or endorsed by the publisher.
