# Supplementary material for: Cleaner production technologies for the amelioration of soil health, biomass and secondary metabolites in Ocimum basilicum L. under Indian Western Himalaya
Source: Front Plant Sci. 2022 Nov 9;13:976295. doi: 10.3389/fpls.2022.976295 (PMC9682627; doi:10.3389/fpls.2022.976295)
Supplement: Supplementary file 1 [file DataSheet_1.doc]

**Table S1.** Soil conditions of experimental field before and after two years consecutive organic biostimulants treatment.

| Attributes | Initial values (Prior experiment) | Final values (After experiment) |
| --- | --- | --- |
| Texture | Silty clay | Silty clay |
| Bulk density (Mg cm^–3^) | 1.39±0.45 | 1.27±0.29 |
| pH | 5.3±0.02 | 5.7±0.01 |
| EC (m mhos cm^–1^) | 0.21±0.19 | 0.27±0.13 |
| CEC (c mol_c_ kg^-1^) | 7.14±0.27 | 7.78±0.32 |
| OC (%) | 0.49±0.11 | 0.64±0.16 |
| TC (g kg^–1^) | 40.13±0.51 | 64.78±0.46 |
| TN (g kg^–1^) | 3.89±0.38 | 3.91±0.33 |
| C/N ratio | 10.32±0.18 | 16.57±0.13 |
| Available N (kg ha^–1^) | 139.10±2.39 | 179.3±1.95 |
| Available P (kg ha^–1^) | 7.60±0.87 | 12.48±1.18 |
| Available K (kg ha^–1^) | 318.00±3.19 | 361.3±2.78 |
| Available Mn (mg kg^–1^) | 9.70±0.07 | 10.4±0.04 |
| Available Fe (mg kg^–1^) | 28.10±0.06 | 29.4±0.05 |
| Available Cu (mg kg^–1^) | 0.63±0.03 | 0.69±0.04 |
| Available Zn (mg kg^–1^) | 0.59±0.02 | 0.64±0.02 |
| SMR (CO_2_ g^–1^ dry soil day^–1^) | 0.69±0.11 | 0.94±0.09 |
| MBC (mg C 100 g^–1^ dry soil) | 37.89±0.24 | 69.71±0.19 |
| BPC (× 10^7^ CFU g^–1^ soil) | 6.93±0.71 | 9.56±0.47 |
| DHA (μg TPF g^−1^ dry soil h^−1^) | 0.89±0.21 | 1.27±0.19 |
| Urease (μg NH4^+^ -N g^−1^ dry soil h^−1^) | 29.37±0.11 | 31.79±0.14 |
| ALP (μg PNP g^−1^ dry soil h^−1^) | 21.76±0.19 | 29.16±0.09 |
| β-glucosidase (μg PNG g^−1^ dry soil h^−1^) | 11.51±0.22 | 16.18±0.17 |

Results are represented as mean of three replications (*n=3*) ± SE. pH: potential of hydrogen; EC: electrical conductivity; CEC : cation exchange capacity; OC: organic carbon; TC: total carbon; TN: total nitrogen; C/N: ratio of TC/TN; N: nitrogen; P: phosphorous; K: potassium; Mn: manganese; Fe: iron; Cu: copper; Zn: zinc; SMR: soil microbial respiration; MBC: microbial biomass carbon; DHA: dehydrogenase activity; ALP: alkaline phosphatase activity; TPF: triphenylformazan; PNP: p-nitrophenyl phosphate; PNG: p-nitrophenyl β-glucoside.

**Table S2. Effect of cropping year, organic manure, biofertilizer, and seaweed extract on photosynthetic pigments and gaseous exchange in *O. basilicum*.**

| Treatments | Chl_a_  (mg g^−1^) | Chl_b_  (mg g^−1^) | Carotenoids  (mg g^−1^) | P_n_  (μmol m^−2^ s^−1^) | G_s_  (mol m^−2^ s^−1^) | C_i_  (µmol mol^−1^) | T_r_  (mmol m^−2^ s^−1^) | VPD_Leaf_  (Kpa) | WUE_int_ | C_i_/C_a_ |
| --- | --- | --- | --- | --- | --- | --- | --- | --- | --- | --- |
| Cropping year |  |  |  |  |  |  |  |  |  |  |
| 2020 | 4.11 b | 2.14 b | 2.27 b | 18.21 b | 0.44 b | 188.72 b | 5.11 | 1.62 b | 3.56 b | 0.47 b |
| 2021 | 4.21 a | 2.25 a | 2.35 a | 22.69 a | 0.63 a | 214.16 a | 6.21 | 1.88 a | 3.65 a | 0.54 a |
| SEm | 0.07 | 0.08 | 0.09 | 0.22 | 0.05 | 2.38 | 0.15 | 0.12 | 0.14 | 0.21 |
| LSD (*P=0.05*) | 0.02 | 0.02 | 0.02 | 0.53 | 0.01 | 5.82 | NS | 0.03 | 0.06 | 0.05 |
| Vermicompost |  |  |  |  |  |  |  |  |  |  |
| V- | 3.97 c | 2.11 c | 2.25 c | 18.63 c | 0.45 c | 190.51 c | 5.29 c | 1.66 c | 3.52 | 0.48 |
| V4 | 4.10 b | 2.21 b | 2.34 ab | 19.48 b | 0.48 b | 203.05 b | 5.37 b | 1.73 b | 3.63 | 0.51 |
| V8 | 4.39 a | 2.27 a | 2.35 a | 21.23 a | 0.57 a | 210.76 a | 5.78 a | 1.85 a | 3.67 | 0.53 |
| SEm | 0.08 | 0.10 | 0.12 | 0.27 | 0.17 | 2.91 | 0.19 | 0.15 | 0.19 | 0.27 |
| LSD (*P=0.05*) | 0.03 | 0.02 | 0.03 | 0.65 | 0.01 | 7.13 | 0.45 | 0.04 | NS | NS |
| Biofertilizer |  |  |  |  |  |  |  |  |  |  |
| B- | 3.83 b | 2.17 b | 2.17 b | 19.01 b | 0.47 b | 195.29 b | 5.43 b | 1.69 b | 3.59 b | 0.49 b |
| B+ | 4.48 a | 2.52 a | 2.45 a | 21.88 a | 0.61 a | 207.59 a | 5.84 a | 1.80 a | 3.75 a | 0.52 a |
| SEm | 0.07 | 0.08 | 0.09 | 0.22 | 0.05 | 2.38 | 0.15 | 0.12 | 0.14 | 0.21 |
| LSD (*P=0.05*) | 0.02 | 0.02 | 0.03 | 0.53 | 0.01 | 5.82 | 0.37 | 0.03 | 0.09 | 0.05 |
| Seaweed extract |  |  |  |  |  |  |  |  |  |  |
| S- | 4.06 b | 2.11 b | 2.24 b | 19.13 b | 0.47 b | 198.86 ab | 5.57 b | 1.71 b | 3.43 b | 0.48 b |
| S+ | 4.25 a | 2.28 a | 2.39 a | 21.06 a | 0.56 a | 204.02 a | 5.70 a | 1.79 a | 3.69 a | 0.51 a |
| SEm | 0.07 | 0.08 | 0.09 | 0.22 | 0.05 | 2.38 | 0.15 | 0.12 | 0.14 | 0.21 |
| LSD (*P=0.05*) | 0.02 | 0.02 | 0.03 | 0.53 | 0.01 | 5.82 | 0.37 | 0.03 | 0.09 | 0.05 |

Results are represented as mean of three replications (*n=3*) of two years pooled data, different lowercase letters in the same column of each treatment are significantly different at (*P=0.05*); SEm: standard error of mean; LSD: least significant difference (*P=0.05*); NS: non-significant; V-: unfertilized control; V4: vermicompost @ 4 Mg ha**^−^**^1^; V8: vermicompost @ 8 Mg ha^−1^; B: biofertilizer; S: seaweed extract @ 7 mL L**^−^**^1^; (-): without factor; (+): with factor; Chl_a_: chlorophyll a; Chl_b_: chlorophyll b; P_n_: net photosynthetic rate; G_s_: stomatal conductance; C_i_: CO_2_ mole fraction in the leaf intercellular air spaces; T_r_: transpiration rate; VPD_Leaf_: leaf vapor pressure deficit; WUE_int_: internal water use efficiency; Ci: intra cellular CO_2_; Ci/Ca: ratio of intracellular to ambient CO_2_.

**Table S3. Effect of cropping year, organic manure, biofertilizer, and seaweed extract on yield and associated attributes in *O. basilicum* at harvest (100 DAT).**

| Treatments | Inflorescence plant^−1^ (100 DAT) | Inflorescence length plant^−1^ (cm) | Leaf FW (g) | Inflorescence FW (g) | Stem FW (g) | Leaf+Inflorescence FW (g) | | Leaf+Inflorescence/stem ratio | Herbage yield (g plant^−1^) | | Herbage yield (Mg ha^−1^) | EO content (% w/w) | Oil yield (kg ha^−1^) |
| --- | --- | --- | --- | --- | --- | --- | --- | --- | --- | --- | --- | --- | --- |
| 2020 | 26.02 b | 17.23 b | 51.52 b | 43.70 b | 40.04 b | 95.22 b | | 2.44 | 135.26 b | | 6.01 b | 0.97 b | 55.03 b |
| 2021 | 39.28 a | 26.05 a | 56.96 a | 53.08 a | 44.37 a | 110.04 a | | 2.53 | 154.41 a | | 6.86 a | 1.05 a | 69.03 a |
| SEm | 0.39 | 0.28 | 0.59 | 0.44 | 0.54 | 0.60 | | 0.05 | 0.84 | | 0.10 | 0.13 | 0.54 |
| LSD (*P=0.05*) | 1.12 | 0.80 | 1.68 | 1.25 | 1.53 | 1.66 | | NS | 2.40 | | 0.11 | 0.04 | 2.42 |
| V- | 28.09 c | 17.75 c | 45.11 c | 32.99 c | 30.20 c | 78.10 c | | 2.63 a | 108.30 c | | 4.81 c | 0.79 c | 34.39 c |
| V4 | 30.99 b | 19.92 b | 51.48 b | 50.35 b | 40.60 b | 101.83 b | | 2.52 ab | 142.43 b | | 6.33 b | 0.98 ab | 57.41 b |
| V8 | 38.88 a | 27.24 a | 66.12 a | 61.83 a | 55.83 a | 127.94 a | | 2.30 bc | 183.77 a | | 8.17 a | 1.03 a | 94.29 a |
| SEm | 0.48 | 0.34 | 0.72 | 0.54 | 0.66 | 0.58 | | 0.06 | 1.03 | | 0.12 | 0.16 | 0.66 |
| LSD (*P=0.05*) | 1.38 | 0.98 | 2.06 | 1.53 | 1.88 | 2.03 | | 0.16 | 2.94 | | 0.13 | 0.05 | 2.96 |
| B- | 28.96 b | 18.80 b | 48.34 b | 42.85 b | 38.52 b | 91.19 b | | 2.39 b | 129.70 b | | 4.76 b | 0.86 b | 46.30 b |
| B+ | 36.35 a | 24.47 a | 60.14 a | 53.93 a | 45.90 a | 114.07 a | | 2.58 a | 159.97 a | | 7.11 a | 1.25 a | 77.76 a |
| SEm | 0.39 | 0.28 | 0.59 | 0.44 | 0.54 | 0.60 | | 0.05 | 0.84 | | 0.10 | 0.13 | 1.88 |
| LSD (*P=0.05*) | 1.12 | 0.80 | 1.68 | 1.25 | 1.53 | 1.66 | 0.13 | | 2.40 | 0.11 | | 0.04 | 2.42 |
| S- | 30.61 b | 20.51 b | 51.37 b | 46.07 b | 40.39 b | 97.44 b | | 2.45 | 137.82 b | | 4.93 b | 0.92 b | 53.63 b |
| S+ | 34.70 a | 22.77 a | 57.11 a | 50.71 a | 44.03 a | 107.82 a | | 2.52 | 151.85 a | | 6.15 a | 1.09 a | 70.43 a |
| SEm | 0.39 | 0.28 | 0.59 | 0.44 | 0.54 | 0.60 | | 0.05 | 0.84 | | 0.10 | 0.13 | 1.88 |
| LSD (*P=0.05*) | 1.12 | 0.80 | 1.68 | 1.25 | 1.53 | 1.66 | | NS | 2.40 | | 0.11 | 0.04 | 2.42 |

Results are represented as mean of three replications (*n=3*) of two years pooled data, different lowercase letters in the same column of each treatment are significantly different at (*P=0.05*); DAT: days after transplantation; FW: fresh weight; EO: essential oil; SEm: standard error of mean; LSD: least significant difference (*P=0.05*); NS: non-significant; V-: unfertilized control; V4: vermicompost @ 4 Mg ha**^−^**^1^; V8: vermicompost @ 8 Mg ha**^−^**^1^; B: biofertilizer; S: seaweed extract @ 7 mL L**^−^**^1^; (-): without factor; (+): with factor.

**Table S4. Effect of cropping year, organic manure, biofertilizer, and seaweed extract on composition (area %) *O. basilicum* EO.**

| Treatments | Hexenyl acetate <(3E)> | Cineole <1,8> | Linalool | Methyl chavicol | Caryophyllane <4,8-epoxy> | Bergamotene <cis> | Cadinene |
| --- | --- | --- | --- | --- | --- | --- | --- |
| RI (E) | 991 | 1048 | 1103 | 1204 | 1423 | 1434 | 1519 |
| RI (L) | 1001 | 1032 | 1102 | 1195 | 1423 | 1432 | 1513 |
| Cropping year |  |  |  |  |  |  |  |
| 2020 | 0.51 b | 0.52 b | 25.17 b | 61.88 b | 0.62 | 0.55 | 1.66 b |
| 2021 | 0.65 a | 0.63 a | 26.60 a | 63.64 a | 0.63 | 0.54 | 2.21 a |
| SEm | 0.07 | 0.13 | 0.19 | 0.13 | 0.13 | 0.11 | 0.08 |
| LSD (*P=0.05*) | 0.09 | 0.09 | 0.53 | 0.37 | NS | NS | 0.23 |
| Vermicompost |  |  |  |  |  |  |  |
| V- | 0.55 bc | 0.56 | 24.66 c | 61.96 c | 0.64 | 0.57 | 1.85 |
| V4 | 0.56 b | 0.57 | 25.90 b | 62.69 b | 0.66 | 0.60 | 1.95 |
| V8 | 0.63 a | 0.59 | 27.09 a | 63.64 a | 0.69 | 0.61 | 2.01 |
| SEm | 0.15 | 0.15 | 0.23 | 0.16 | 0.17 | 0.14 | 0.10 |
| LSD (*P=0.05*) | 0.02 | NS | 0.65 | 0.45 | NS | NS | NS |
| Biofertilizer |  |  |  |  |  |  |  |
| B- | 0.54 b | 0.59 | 24.81 b | 61.75 b | 0.65 | 0.57 b | 1.85 |
| B+ | 0.62 a | 0.56 | 26.96 a | 63.78 a | 0.68 | 0.61 a | 2.02 |
| SEm | 0.07 | 0.12 | 0.19 | 0.13 | 0.14 | 0.11 | 0.08 |
| LSD (*P=0.05*) | 0.09 | NS | 0.53 | 0.37 | NS | 0.03 | NS |
| Seaweed extract |  |  |  |  |  |  |  |
| S- | 0.56 ab | 0.60 | 25.42 b | 62.16 b | 0.66 | 0.58 | 1.88 |
| S+ | 0.60 a | 0.56 | 26.35 a | 63.36 a | 0.67 | 0.60 | 2.00 |
| SEm | 0.07 | 0.12 | 0.19 | 0.13 | 0.14 | 0.11 | 0.08 |
| LSD (*P=0.05*) | 0.09 | NS | 0.53 | 0.37 | NS | NS | NS |

Results are represented as mean of three replications (*n=3*) of two years pooled data, different lowercase letters in the same column of each treatment are significantly different at (*P=0.05*); EO: essential oil; SEm: standard error of mean; RI (E): experimental retention indices; RI (L): retention indices provided in literature; LSD: least significant difference (*P=0.05*); NS: non-significant; V-: unfertilized control; V4: vermicompost @ 4 Mg ha**^−^**^1^; V8: vermicompost @ 8 Mg ha**^−^**^1^; B: biofertilizer; S: seaweed extract @ 7 mL L**^−^**^1^; (-): without factor; (+): with factor.

**Table S5.** Interaction effect of organic manure, biofertilizers, and seaweed extract on herbage and EO yield of *O. basilicum*.

| Herbage yield (Mg ha**^−^**^1^) | | | | | | | | | | | | | | | | | | | | | | | | | |
| --- | --- | --- | --- | --- | --- | --- | --- | --- | --- | --- | --- | --- | --- | --- | --- | --- | --- | --- | --- | --- | --- | --- | --- | --- | --- |
| 2020 | | | | | | | | | | | | 2021 | | | | | | | | | | | | | |
| Treatment | | B- | | | B+ | | | | | B- | | | | | | | B+ | | |  | | |  | | |
|  |  | S- | | S+ | S- | | S+ | | | | | | S- | S+ | S- | | | | S+ | Mean (V) | |  | | | |
| V- | | 4.48 | | 4.53 | 4.66 | | 4.89 | | | | | | 4.19 | 4.80 | 5.31 | | | | 5.64 | 4.81 | |  | | | |
| V2 | | 5.43 | | 5.59 | 6.02 | | 6.41 | | | | | | 5.80 | 6.35 | 7.13 | | | | 7.91 | 6.33 | |  | | | |
| V3 | | 6.57 | | 7.12 | 7.56 | | 8.86 | | | | | | 6.69 | 7.62 | 9.66 | | | | 11.25 | 8.17 | |  | | | |
| Mean (Y) | | 6.01 | | | | | | | | | | 6.86 | | | | | | | |  | | | | |  |
| Mean (B) | | 5.76 | | | 7.11 | | | | |  | | | | | | | | | |  | | |  | | |
| Mean (S) | | 6.13 | | | 6.75 | | | | | LSD (V×B) | | | | | | | | | | 4.70 | | |  | | |
| LSD (Y) | | 13.03 | |  |  | | |  | | | | | LSD (V×S) | | | | | | | 1.04 | | | |  | |
| LSD (V) | | 67.71 | |  |  | | |  | | | | | LSD (B×S) | | | | | | | 0.38 | | | |  | |
| LSD (B) | | 32.57 | |  |  | | |  | | | | | LSD (Y×V×B×S) | | | | | | | 0.02 | | | |  | |
| LSD (S) | | 6.99 | |  |  | | |  | | | | | LSD (Y×V×B) | | | | | | | 0.73 | | | |  | |
| LSD (Y×V) | | 1.35 | |  |  | | |  | | | | | LSD (Y×V×S) | | | | | | | 0.001 | | | |  | |
| LSD (Y×B) | | 5.74 | |  |  | | |  | | | | | LSD (Y×B×S) | | | | | | | 0.04 | | | |  | |
| LSD (Y×S) | | 0.56 | |  |  | | |  | | | | | LSD (V×B×S) | | | | | | | 0.21 | | | |  | |
| Oil yield (kg ha**^−^**^1^) | | | | | | | | | | | | | | | | | | | | | | | | | |
| 2020 | | | | | | | | | | | | 2021 | | | | | | | | | | | | | |
| Treatment | B- | | | | B+ | | | | | | B- | | | | | | B+ | | |  | | |  | | |
|  | S- | | S+ | | S- | S+ | | | | | | | S- | S+ | | S- | | S+ | | Mean (V) |  | | | | |
| V0 | 27.87 | | 28.95 | | 31.78 | 37.07 | | | | | | | 27.27 | 32.82 | | 38.47 | | 50.90 | | 34.39 |  | | | | |
| V2 | 35.01 | | 41.26 | | 50.44 | 71.30 | | | | | | | 41.21 | 49.16 | | 76.75 | | 94.19 | | 57.41 |  | | | | |
| V3 | 51.44 | | 80.28 | | 91.19 | 113.78 | | | | | | | 49.78 | 90.59 | | 122.39 | | 154.87 | | 94.29 |  | | | | |
| Mean (Y) | 55.03 | | | | | | | | 69.03 | | | | | | | | | | |  | | |  | | |
| Mean (B) | 46.30 | | | | 77.76 | | | | | |  | | | | | | | | |  | | |  | | |
| Mean (S) | 53.63 | | | | 70.43 | | | | | | LSD (V×B) | | | | | | | | | 4.18 | | |  | | |
| LSD (Y) | 2.42 | |  | |  | | |  | | | | | LSD (V×S) | | | | | | | 3.41 | | | | |  |
| LSD (V) | 2.96 | |  | |  | | |  | | | | | LSD (B×S) | | | | | | | 2.17 | | | | |  |
| LSD (B) | 2.42 | |  | |  | | |  | | | | | LSD (Y×V×B×S) | | | | | | | NS | | | | |  |
| LSD (S) | 2.42 | |  | |  | | |  | | | | | LSD (Y×V×B) | | | | | | | 5.92 | | | | |  |
| LSD (Y×V) | 4.18 | |  | |  | | |  | | | | | LSD (Y×V×S) | | | | | | | NS | | | | |  |
| LSD (Y×B) | 3.42 | |  | |  | | |  | | | | | LSD (Y×B×S) | | | | | | | NS | | | | |  |
| LSD (Y×S) | NS | |  | |  | | |  | | | | | LSD (V×B×S) | | | | | | | 5.92 | | | | |  |

Results are represented as mean of three replications (*n=3*) of two years pooled data. Y: cropping year; V: vermicompost; B: biofertilizer; S: seaweed extract, combination of letters represent interaction in between; NS: non-significant; LSD: least significant difference (*P=0.05*).
