# Supplementary material for: Cleaner production technologies for the amelioration of soil health, biomass and secondary metabolites in Ocimum basilicum L. under Indian Western Himalaya
Source: Front Plant Sci. 2022 Nov 9;13:976295. doi: 10.3389/fpls.2022.976295 (PMC9682627; doi:10.3389/fpls.2022.976295)
Supplement: Supplementary file 2 [file DataSheet_2.doc]

|  |
| --- |
| **Supplementary Figure S1**. Thirty days old seedlings (30 DOS) of Indian sweet basil used for transplanting. (a) Uninoculated control seedings; (b) Seedings treated with biofertilizer |

|  |
| --- |
| **Supplementary Figure S2**. Light response curve in *O. basilicum* under the western Himalayan condition |
